# Neural signatures of engagement in driving: comparing active control and passive observation

**DOI:** 10.3389/fnins.2025.1698625

**Published:** 2025-11-06

**Authors:** Zixin Li, Hiroyuki Kambara, Yasuharu Koike

**Affiliations:** 1 Department of Information and Communications Engineering, Institute of Science Tokyo, Yokohama, Japan; 2 Information Technology Course, Faculty of Engineering, Tokyo Polytechnic University, Atsugi, Japan; 3 Institute of Integrated Research, Institute of Science Tokyo, Yokohama, Japan

**Keywords:** electroencephalography (EEG), neural engagement, driving simulation, brain machine interface (BMI), manual vs. automated driving, source localization, cognitive load

## Abstract

Understanding how the human brain differentiates between active engagement and passive observation is a fundamental question in cognitive neuroscience. Using a matched-stimulus driving paradigm to isolate engagement from sensory input, we recorded whole-brain EEG while participants performed a manual control task and passively viewed a replay of their own performance. Manual control elicited distinct spectral signatures, including stronger frontal midline theta power and, paradoxically, greater occipital alpha power, consistent with heightened cognitive control and active attentional filtering. While a classifier could distinguish these states with high within-subject accuracy, performance declined in cross-subject validation, highlighting inter-individual variability. These findings delineate the distinct neural signatures of active versus passive engagement under controlled conditions. This work establishes a foundational neurophysiological baseline that can inform research on cognitive state monitoring and the design of neuroadaptive systems in complex human-machine interaction.

## 1 Introduction

Humans frequently alternate between active interaction with their environment and passive observation of external events. These shifts in engagement may appear effortless but are supported by distinct neural mechanisms. Understanding how the brain differentiates between these engagement modes and how it manages transitions between them is a fundamental question in cognitive neuroscience and neuroengineering.

Contemporary theoretical frameworks suggest that active control requires top-down cognitive processes, including ongoing model updating, error monitoring, and attentional allocation. In contrast, passive states are associated with reduced executive activity and diminished sensory processing. Predictive coding theory posits that the brain minimizes prediction errors by continuously refining internal models through perception and action (Friston, 2010). Active behavior reflects this process of inference, while passive states permit predictive stability with minimal cognitive demand. Additionally, cognitive load theory indicates that task complexity modulates neural resource allocation, such that more demanding tasks require greater executive control (Borghini et al., 2014; Gevins et al., 1997). Accordingly, predictive coding provides a unifying framework for our hypotheses: manual driving requires continuous prediction updating and error minimization, which should manifest as elevated frontal theta and stronger fronto-parietal engagement. By contrast, passive replay stabilizes predictions with minimal updating demands, leading to attenuated executive activity and reduced reliance on occipito-parietal alpha gating.

Electroencephalography offers well-characterized spectral markers that map onto core control and attention processes in dynamic tasks. Frontal midline theta (4–8 Hz) indexes cognitive control and conflict/error monitoring and reliably scales with control demands and proactive/reactive regulation in fronto-parietal circuits (Cavanagh and Frank, 2014; Eisma et al., 2021). Occipital–parietal alpha (8–12/13 Hz) reflects attentional selection: alpha desynchronization accompanies enhanced sensory processing, whereas alpha synchronization implements top-down inhibitory gating of task-irrelevant input (Clayton et al., 2015; Jensen, 2024; Jensen and Mazaheri, 2010; Klimesch, 2012). In the sensorimotor system, mu (8–13 Hz) and beta (13–30 Hz) track motor preparation and sensorimotor integration–mu ERD and beta modulation accompany movement execution and predictive control (Pfurtscheller and Lopes da Silva, 1999; Pfurtscheller and Neuper, 1997). These markers have been leveraged to index engagement in realistic settings, including driving paradigms (Borghini et al., 2014; Lin et al., 2008; Walshe et al., 2023), and provide a principled basis for our hypotheses linking manual control to elevated frontal-theta (control allocation) and mode-dependent alpha dynamics (attentional gating), alongside motor-band changes related to action readiness.

In the present matched-stimulus design, we therefore predicted stronger frontal-midline theta during manual control (reflecting greater control allocation) and systematic alpha modulation in occipital–parietal cortices (reflecting top-down filtering) relative to passive replay, with mu/beta changes indexing sensorimotor demands. Consistent with these band-specific roles, fronto-parietal coupling in theta/beta ranges is also implicated in coordinating executive control with visuospatial processing during naturalistic action (Cooper et al., 2015).

Despite this evidence, previous studies have often confounded engagement mode with sensory input. For example, comparing manual control with passive video observation introduces uncontrolled differences in visual motion, vestibular input, and auditory cues. These factors limit interpretability and reduce ecological validity. Furthermore, real-world studies have reported mixed effects of automation on engagement, influenced by task realism, interface design, and individual differences (Merat et al., 2014; Parasuraman and Manzey, 2010).

The objective of this study is to identify and characterize the neurophysiological signatures of active control and passive observation under matched sensory conditions, and to quantify the cognitive demands of transitioning between these modes. To achieve this, we employed a matched-replay paradigm in which participants completed a manual driving task and then passively viewed a replay of their performance. Because visual stimuli were identical across conditions, any differences in brain activity can be attributed to engagement mode rather than sensory variance.

We analyzed electroencephalographic data using a multi-layered approach. First, we evaluated spectral dynamics, focusing on frontal theta and occipital alpha as indicators of cognitive control and visual attention. Second, we assessed functional connectivity to examine how brain networks reorganize across conditions (Bastos et al., 2015). Third, we applied machine learning to test whether these neural signatures can support accurate classification of cognitive state (Lotte et al., 2007; Roy et al., 2019).

Our findings indicate that manual control is associated with increased frontal theta power, greater occipital alpha power, and enhanced fronto-parietal connectivity, reflecting effortful, goal-directed engagement. In contrast, passive observation is characterized by a more modular, disengaged network state. These differences are amplified under higher task complexity, suggesting that re-engagement from passive to active control imposes measurable cognitive demands (Neubauer et al., 2012; Parasuraman and Manzey, 2010).

By establishing a clear neurophysiological distinction between engagement modes under tightly controlled conditions, this study provides a foundation for future work on dynamic cognitive state monitoring. It also informs the design of neuroadaptive systems that respond appropriately to fluctuations in human engagement during complex tasks.

## 2 Materials and methods

### 2.1 Participants

Eleven healthy adults (8 males, 3 females; mean age = 25.9 ± 2.7 years, range: 22–30) participated in the study. All had normal or corrected-to-normal vision and no history of neurological or psychiatric conditions. Nine participants reported real-world driving experience (mean = 4.8 ± 2.8 years), and five had prior exposure to driving simulation games; this background information was collected for exploratory purposes. All procedures were approved by the Institutional Review Board of the Institute of Science Tokyo and conducted in accordance with the Declaration of Helsinki. Written informed consent was obtained from all participants, who were recruited from the university community and received monetary compensation for their participation.

### 2.2 Experimental design and procedure

#### 2.2.1 Driving simulator setup

The experiment was conducted using the commercial racing simulation software Assetto Corsa: Competizione on the Monza racing circuit. This software is widely used in research and training contexts because it provides realistic vehicle dynamics, high-fidelity track environments, and a replay function that allows identical visual stimuli to be presented across active and passive conditions. These features made it well-suited for isolating engagement effects while maintaining ecological validity. The simulator setup included a steering wheel and pedal system mounted on a racing seat to provide realistic control and immersion. Visual stimuli were presented on a widescreen monitor positioned at eye level. A video camera recorded both the participant and the driving scene to enable behavioral analysis and synchronization with EEG recordings. All sessions were carried out in a controlled laboratory environment. To minimize environmental confounds, the room was kept quiet with curtains closed to block external light sources, and the monitor brightness and contrast were fixed across sessions. Ambient illumination and temperature were maintained constant, and participants were seated at a fixed distance from the screen to ensure consistent visual conditions.

#### 2.2.2 Experimental conditions

A 2 *(Driving Mode: Manual vs. Automated (AD - replay)) × 2 (Task Complexity: Easy vs. Hard)* within-subjects factorial design was used.

In the Manual Driving (MD) condition, participants actively controlled the vehicle’s speed and steering using a racing simulator while completing laps on the Monza circuit. In the Automated Driving - Passive Replay (AD-replay) condition, participants viewed a replay of their own preceding MD session on the same setup, adopting the role of a supervisory passenger without any control authority. This framing reflects common terminology in automated driving research, emphasizing monitoring, situational awareness, and takeover readiness. Participants were instructed to place both hands on their knees and keep their feet away from the pedals, refraining from touching the steering wheel or performing any overt driving-related actions. At the same time, because the replay reflected their own prior driving trajectory, they were encouraged to remain mentally engaged with the unfolding task, monitoring the traffic scene and anticipating upcoming curves as if overseeing an automated driving system. In this sense, motor imagery was neither explicitly required nor prohibited, but participants were asked to maintain an engaged supervisory stance rather than a purely passive viewing mode. The experimenter monitored participants throughout the AD-replay session to ensure compliance with these instructions.

**FIGURE 1:**
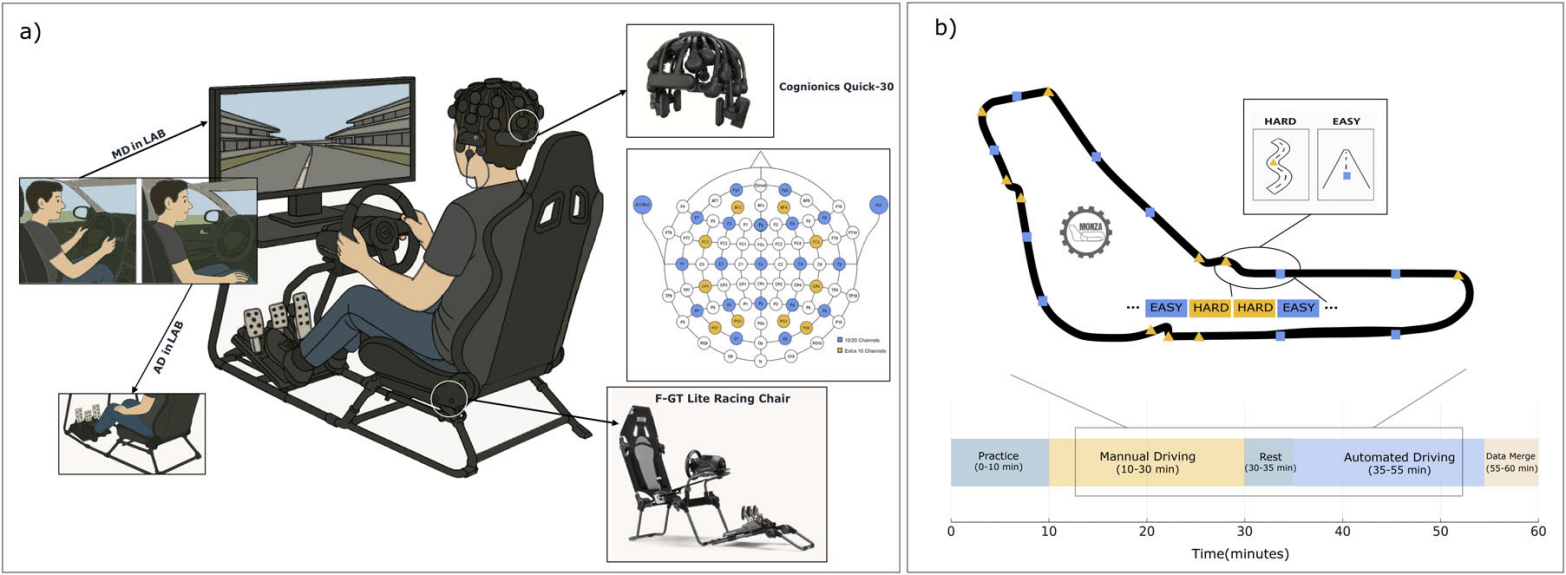
Experimental setup and protocol for EEG-based driving simulation. **(a)** Illustration of the driving simulation environment. Participants used a realistic racing simulator (Assetto Corsa: Competizione), consisting of an F-GT lite racing chair, steering wheel, and pedal controls. EEG data were recorded using a Cognionics Quick-30 wireless dry-electrode headset with 29 scalp channels; two earlobe electrodes (A1/A2) served as online reference (31 electrodes in total). Analyses used the 29 scalp channels. The inset details the electrode layout. Two distinct modes were employed: Manual Driving (MD), in which participants actively engaged with the controls, and Automated Driving - Passive Replay (AD-replay), in which participants passively monitored a replay of their own previous driving session without physical interaction. **(b)** Depiction of the Monza circuit layout, delineating the predefined “EASY” and “HARD” complexity segments. Segment markers were synchronized with EEG recordings. The bottom timeline illustrates the experimental protocol: Practice, MD, AD-replay, and Data Merge/Rest phases. EEG data from both modes (MD and AD-replay) and both complexities (EASY and HARD) were analyzed to differentiate cognitive states associated with active control and passive monitoring.

This replay approach ensured identical visual input across conditions (see Section “2.2.3 Experimental task and procedure”). Task complexity was defined based on track geometry, with segment labels manually annotated during the MD session and reused for the AD-replay playback. This classification followed prior evidence indicating that curved roads impose greater cognitive and sensorimotor demands than straight segments due to the need for continuous steering, speed regulation, and spatial monitoring (Mecheri et al., 2022; Vos et al., 2023; Wascher et al., 2018). And the Monza circuit is a classic motorsport track frequently used in Formula 1 and GT racing, and its geometry is well documented. This allowed us to reference established track data when defining Easy (straight) and Hard (curved) segments, facilitating objective validation of our manual annotations. Easy segments consisted of long, straight sections requiring minimal steering and modest acceleration, while Hard segments involved sharp or sustained curves necessitating braking, precise control, and heightened attentional engagement. Each lap included a predefined sequence of Easy and Hard segments (approximately 10 versus 10 per lap, respectively). Segment onset and offset markers indicating transitions between Easy and Hard segments were manually annotated based on track geometry and precisely synchronized with EEG recordings. The track segmentation and synchronization procedure are illustrated in Figure 1b.

#### 2.2.3 Experimental task and procedure

Upon arrival, participants were verbally briefed on the experimental procedure, completed a driving history questionnaire, and provided written informed consent. They then completed a supervised practice session in the Manual Driving (MD) mode (10–20 min) to ensure familiarity with the simulator and consistent lap performance. Following practice, EEG preparation was completed (see Section “2.3 EEG data acquisition”), and continuous EEG and synchronized video recording commenced.

The experiment began with the MD session, during which participants drove 4–7 laps (∼15–20 min) while a researcher manually inserted event markers denoting the onset and offset of predefined Easy and Hard segments. After a 5 to 10-min rest, participants completed the AD-replay session by viewing a playback of their own MD run. Consistent with the instructions described in Section “2.2.2 Experimental conditions,” they were asked to adopt the role of supervisory passengers without control authority, to monitor the replay as if overseeing an automated driving system, and to remain mentally engaged while refraining from any overt driving-related actions (e.g., touching the wheel or pedals). The experimenter monitored participants to ensure compliance.

This AD-replay paradigm, based on passive replay of self-driven sessions, was designed to maximize control over visual and temporal stimulus properties while inducing a cognitive stance closer to supervisory monitoring than to purely passive viewing. This allowed the isolation of neural activity differences attributable to driver engagement mode (active vs. supervisory observation) while holding sensory input constant. We note that this approach does not fully emulate the cognitive demands of SAE Level 2–3 automated driving (e.g., continuous system monitoring or real takeover readiness). Rather, it represents a minimal-control, supervisory baseline against which the neural correlates of active manual driving can be compared under varying complexity.

The complete experimental setup–including the driving simulator configuration, EEG acquisition system, driving modes (MD vs. AD-replay), electrode layout, track segmentation, and experimental timeline–is illustrated comprehensively in Figure 1.

### 2.3 EEG data acquisition

Electroencephalography signals were recorded from twenty-nine scalp electrodes arranged according to an extended 10–20 electrode montage, with 2 ear electrodes serving as the online reference during acquisition. Electrode placement corresponding to the Cognionics Quick-30 wireless EEG headset is illustrated in Figure 1a. Data were sampled at 500 Hz using CGX Acquisition software.

Prior to recording, electrode impedance was monitored in real time and adjusted to remain within the manufacturer’s recommended range (200–400 kΩ) (Chi et al., 2012; Lopez-Gordo et al., 2014). The CGX Acquisition software also provided automatic bad-channel flagging, which was monitored during preparation to ensure stable signals. Continuous EEG data were collected throughout the entire duration of the driving tasks.

### 2.4 EEG data preprocessing

Electroencephalography preprocessing was conducted in MATLAB using the EEGLAB toolbox (v2023.1; Swartz Center for Computational Neuroscience, UCSD) (Delorme and Makeig, 2004). Continuous recordings from MD and AD-replay sessions were segmented to exclude non-task periods, retaining approximately 15–18 min of data per session. Event markers corresponding to Easy and Hard segments were synchronized with each participant’s EEG data. Standard 10–20 coordinates were assigned to the 29-scalp-channel Cognionics system (Cognionics Inc., San Diego, CA, USA), and for source-level analysis, electrode positions were co-registered to a standard template; analyses were limited to the 29 recorded scalp EEG channels. Signals were re-referenced to the average of the A1 and A2 earlobe electrodes, and band-pass filtered between 2 and 36 Hz using a finite impulse response (FIR) filter to remove drift and noise. Independent Component Analysis (ICA) was performed using the Infomax algorithm, and components were classified with ICLabel (Pion-Tonachini et al., 2019). Only those with greater than 80% probability of reflecting brain activity were retained; artifactual components (e.g., eye movements, muscle, cardiac) were removed prior to signal reconstruction. To further assess the potential impact of residual artifacts, we quantified the number of ocular and muscle components across conditions and evaluated classifier robustness under different ICA retention thresholds; detailed results are provided in Supplementary Tables 1, 2.

### 2.5 EEG data analysis

#### 2.5.1 Source localization analysis

Cortical source localization was performed using distributed source modeling implemented in MNE-Python (v1.9.0) (Gramfort et al., 2013). EEG data were re-referenced to the average reference by computing projection operators to improve inverse solution stability. Epochs spanning −0.4 to 1.6 s relative to the onset of Easy and Hard segments were extracted and baseline-corrected using the −0.2 to 0 s window. Standard 10–20 electrode locations (29 scalp channels with 2 reference channels) were used. (Electrodes: Fp1/2, AF3/4, F3/z/4/7/8, FC5/6, C3/z/4, T7/8, CP5/6, P3/z/4/7/8, PO3/4/7/8, O1/2). This configuration provides coverage across frontal, central, temporal, parietal, and occipital regions, and largely overlaps with commonly used 32-channel montages, with the exception of some midline/posterior sites (e.g., Oz, POz, CP1, CP2) and the inclusion of additional lateral–posterior sites (PO3, PO4, PO7, PO8). Prior work has shown that while high-density EEG (64–128 channels) improves fine-grained localization, distributed inverse solutions such as dSPM yield robust and interpretable results with moderate-density montages (25–32 channels), where the main accuracy gain occurs compared to sparse (<20) arrays (Hedrich et al., 2017; Mahjoory et al., 2017; Song et al., 2015). Given that our analyses focused on condition-dependent contrasts at the group level rather than millimeter-accurate single-dipole localization, the use of 29 channels is appropriate. Moreover, this portable system was selected to balance source estimation accuracy with experimental feasibility, allowing shorter preparation time and greater participant comfort during prolonged simulated driving sessions.

In the absence of individual MRIs, electrode positions from the extended 10–20 montage (29 scalp channels with 2 references) were mapped to the fsaverage template using MNE’s rigid transform (trans = “fsaverage”) based on nasion/LPA/RPA fiducials; alignment was visually inspected. Template-based co-registration introduces systematic localization uncertainty due to inter-subject anatomical variability. For 10–20 layouts, group studies report cortical projection variability on the order of several millimeters (typically within ∼5–10 mm); for denser 10–10 layouts, comparable per-axis dispersions in this range have also been observed. We therefore treat anatomical labels as putative regional estimates rather than precise loci (e.g., putative mPFC/ACC; putative PPC) (Koessler et al., 2009; Okamoto et al., 2004).

The forward model was constructed based on the fsaverage template and a three-layer Boundary Element Model (BEM) accounting for scalp, skull, and brain conductivities. Unless otherwise noted, tissue conductivities followed standard values (scalp = 0.30 S/m, skull = 0.006 S/m, brain = 0.30 S/m; skull-to-brain ≈ 1:50). The cortical source space was defined using *oct6* spacing, yielding approximately 10,242 source locations per hemisphere. (For reference, an oct-6 grid corresponds to ∼8,196 dipoles in total–∼4,098 per hemisphere–with orientations constrained normal to the cortex; our analyses used the cortex-normal component). EEG sensor positions were co-registered to the head model via a standard transformation. The EEG forward operator (29 channels) had a condition number of ∼4.8 × 10^1^, indicating good numerical conditioning under this head model.

Noise covariance matrices were estimated from the baseline period for each participant and condition. The inverse operator was computed using these covariances and the forward model. Source activity was reconstructed using dynamic statistical parametric mapping (dSPM), incorporating a loose orientation constraint (looseness = 0.2), depth weighting (0.8), and an assumed signal-to-noise ratio (SNR) of 3.0 (λ^2^ = 1/SNR^2^) (Numerically, SNR = 3 implies λ^2^ = 0.111). The resulting inverse solutions were applied to each epoch, and only the component of activity normal to the cortical surface was retained. Source time courses (STCs) were averaged across epochs within each condition (MD-Easy, MD-Hard, AD-replay-Easy, AD-replay-Hard) to generate participant-level estimates for group-level analysis. Given the montage and template anatomy, anatomical labels are reported as putative regional estimates (e.g., putative mPFC/ACC; putative PPC) rather than millimeter-accurate loci; reported spatial resolution in similar EEG settings is on the order of ∼10–15 mm for superficial cortex and ∼20–30 mm for deeper sources (Lantz et al., 2003). To provide converging evidence independent of inverse assumptions, we additionally report sensor-space topographies (condition means and contrasts; Supplementary Figure 3, Sensor-space topographies for all contrasts), which qualitatively corroborate the source-level patterns (e.g., frontal/occipital increases during MD, posterior/parietal increases during AD).

#### 2.5.2 Group-level source activity and region of interest (ROI) analysis

To assess population-level cortical dynamics and test the study’s hypotheses, participant-level average Source Time Courses (STCs; see Section “2.5.1 Source localization analysis”) were aggregated to generate grand-average STCs for each of the four conditions (MD-Easy, MD-Hard, AD-replay-Easy, AD-replay-Hard). Contrast STCs were computed to isolate effects of driving mode and task complexity: MD-Easy minus AD-replay-Easy, MD-Hard minus AD-replay-Hard, MD-Hard minus MD-Easy, and AD-replay-Hard minus AD-replay-Easy. These contrast maps were used to identify condition-specific differences in cortical activation.

For examination of the anatomical specificity of these effects, a region-of-interest (ROI) analysis was conducted. ROIs were defined based on the Desikan-Killiany atlas on the fsaverage cortical template and included the Motor Cortex (precentral gyrus, paracentral lobule, supplementary motor area), Frontal Midline (superior, middle, and medial frontal gyri), Occipital Cortex (e.g., cuneus, lingual gyrus), and Posterior Parietal Cortex (PPC; superior and inferior parietal lobules). For each contrast, mean dSPM values within each ROI were extracted over time using MNE-Python, yielding condition-specific ROI time courses for further statistical testing (see Section “2.6 Statistical analysis”).

Analyses focused on the 0.1–0.8 s window following segment onset, selected to capture cognitive processes relevant to task engagement. This interval encompasses several well-documented functions. Error and conflict monitoring typically emerge within 100–300 ms in medial frontal regions, reflected in early negative deflections such as the ERN/N2 (Botvinick et al., 2001; Overmeyer et al., 2021; Ridderinkhof et al., 2004). Compensatory visual processing unfolds from approximately 150 ms onward, including occipital P1/N1 components that index sensory updating (Luck and Vogel, 1997). Attention modulation in parietal and frontal networks is recruited around 200–500 ms, reallocating resources under changing task demands (Corbetta and Shulman, 2002), and more recent reviews highlight sustained visual attention mechanisms in similar time frames (Huang et al., 2023). Executive control processes such as goal maintenance and top-down regulation are expressed in midfrontal theta activity across 200–600 ms (Cavanagh and Frank, 2014; Miller and Cohen, 2001). Finally, visuospatial attention is strongly engaged within 300–600 ms in parietal and occipital cortices to support spatial tracking and sensorimotor integration (Corbetta and Shulman, 2002; Tosoni et al., 2023). Taken together, these temporal landmarks justify our choice of the 0.1–0.8 s analysis window, as it captures the cascade of perceptual, attentional, and executive operations most critical for differentiating engagement states.

**FIGURE 2:**
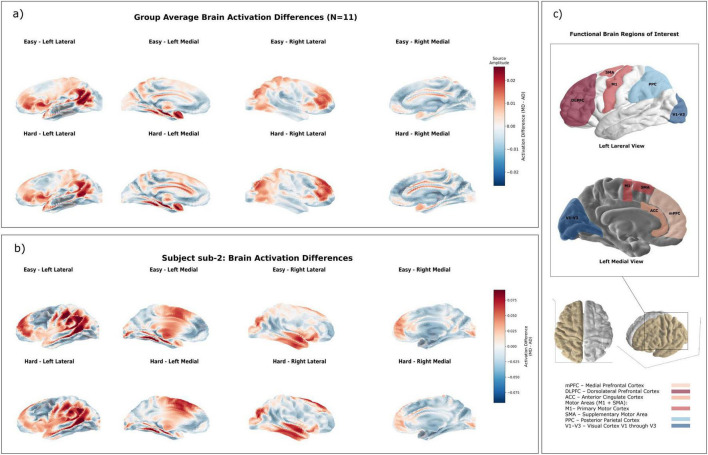
Cortical activation differences between manual and automated driving. **(a)** Group-averaged cortical activation maps (MD minus AD-replay) under easy (top) and hard (bottom) road conditions, visualized from lateral and medial views. Red and blue indicate stronger activity during manual and automated driving, respectively. Values reflect mean dSPM (z-scored SNR) across epochs. **(b)** Example participant (sub-2) showing spatially consistent activation patterns with the group results, including increased mPFC and occipital activity during MD and enhanced parietal activity during AD-replay. **(c)** Schematic of the left hemisphere displaying the functional ROIs examined in this study. Lateral and medial views highlight mPFC, DLPFC, ACC, M1, SMA, PPC, and V1–V3. ROI abbreviations and colors match those used in statistical analyses. Whole-brain maps are shown in arbitrary units (a.u.) for descriptive visualization.

Beyond these procedural definitions, it is also important to clarify the rationale for our ROI grouping strategy. The ROI definitions were theory-driven and tailored to the primary aim of identifying neural signatures of driving engagement. Frontal midline and motor regions were emphasized for mode contrasts (MD vs. AD-replay), whereas posterior parietal and prefrontal regions were prioritized for task complexity (Hard vs. Easy). Although finer atlas-based subdivisions (e.g., M1 vs. SMA, V1 vs. V2–V3) could in principle be analyzed separately (see Figure 2c), we aggregated them into six functionally coherent ROIs. This grouping reduces the multiple-comparison burden and increases statistical power given our modest sample size (*N* = 11), while remaining consistent with prior EEG/neuroimaging work emphasizing functional groupings such as frontal midline theta, motor μ/β, and occipital α. Importantly, each functional ROI was derived from anatomically validated subdivisions (e.g., Motor ROI included precentral gyrus, paracentral lobule, SMA; Occipital ROI included cuneus and lingual gyrus), ensuring anatomical validity while allowing hypothesis-driven functional grouping.

To further substantiate this choice, we conducted internal consistency checks across subregions (e.g., M1 vs. SMA, V1–V3, dlPFC vs. vmPFC). These subregional consistency analyses are visualized in Supplementary Figure 1, which summarizes (a) visual cortex (V1–V3) MD vs. AD-replay contrasts showing consistent activation trends, and (b) frontal midline (dlPFC vs. vmPFC) responses exhibiting comparable complexity effects across engagement modes. We validated regional groupings using three complementary metrics: (1) directional agreement of activation effects (>80% threshold), (2) inter-subregional temporal correlations (*r* > 0.5), and (3) one-way ANOVA testing for subregional differences (*p* > 0.05 indicating homogeneity). The results indicated that subregions within each broader ROI exhibited highly similar activation trends, significant temporal correlations, and no systematic differences in ANOVA testing. This convergence supports the robustness of the chosen grouping scheme and confirms that the broader ROIs adequately capture the complexity effects without obscuring meaningful subregional divergence. While finer parcellations may provide additional insights in larger cohorts or multimodal studies, the present grouping strikes the most appropriate balance between anatomical specificity, statistical robustness, and interpretability for the current research aims.

#### 2.5.3 Frequency domain analysis (power and ERD/ERS)

Frequency-domain analyses were conducted on ROI time courses derived from group-level contrast STCs (see Section “2.5.2 Group-level source activity and region of interest (ROI) analysis”) to investigate oscillatory activity associated with driving mode and task complexity. Four canonical EEG frequency bands were analyzed: Theta (4–7 Hz), Alpha (8–12 Hz), Mu (8–13 Hz, specific to the Motor Cortex), and Beta (13–30 Hz), based on their relevance to cognitive and sensorimotor processes (Klimesch, 1999; Pfurtscheller and Lopes da Silva, 1999). For each ROI and contrast condition (e.g., MD-Easy minus AD-replay-Easy), two metrics were computed within the 0.1–0.8 s post-segment window: (1) band power, calculated as the mean squared amplitude of the band-pass filtered signal, and (2) event-related desynchronization/synchronization (ERD/ERS), expressed as the percentage change in power relative to a pre-segment baseline (−0.2 to 0 s), with negative and positive values reflecting desynchronization and synchronization, respectively (Pfurtscheller and Lopes da Silva, 1999).


$$ERD/ERS\left(\%\right)=\frac{\left(P\,o\,w\,e\,r_{a\,c\,t\,i\,v\,e}-P\,o\,w\,e\,r_{b\,a\,s\,e\,l\,i\,n\,e}\right)}{P\,o\,w\,e\,r_{b\,a\,s\,e\,l\,i\,n\,e}}\times 100$$

These metrics were computed for predefined ROI–band combinations aligned with the study’s hypotheses: Beta and Mu bands in the Motor Cortex for sensorimotor control (Pfurtscheller and Neuper, 1997); Alpha in the Occipital Cortex for visual processing (Klimesch, 1999); Theta in the Frontal Midline for executive control and conflict monitoring (Cavanagh and Frank, 2014); and Alpha in the Posterior Parietal Cortex for visuospatial attention (Jensen and Mazaheri, 2010). The resulting values were subjected to statistical analysis as described in Section “2.6 Statistical analysis.”

#### 2.5.4 Functional connectivity analysis

To evaluate functional interactions between cortical regions, we computed pairwise connectivity metrics between ROI time courses (see Section “2.5.2 Group-level source activity and region of interest (ROI) analysis”). Specifically, Pearson correlation coefficients were calculated to index linear inter-regional coupling,


$$r_{x\,y}=\frac{c\,o\,v\,\left(x,y\right)}{\sigma_{x}\,\sigma_{y}}$$

and magnitude-squared coherence was computed to capture frequency-specific synchronization across ROIs,


$$C_{xy}(f)=\frac{|P_{xy}(f){|}^{2}}{P_{xx}\;(f)\;P_{yy}\;(f)}$$

where *x*(*t*) and *y*(*t*) denote ROI time courses, σ indicates standard deviation, and *P*_*xx*_ (*f*), *P*_*yy*_ (*f*), *P*_*xy*_ (*f*) denote auto- and cross-spectral density functions. Connectivity analyses were implemented in Python using SciPy and MNE-Python.

Because EEG connectivity measures are inherently sensitive to volume conduction and field spread, our approach mitigated these effects in two ways: (1) analyses were performed at the source level after dSPM projection, which reduces spurious correlations driven by common sensor projections (Mahjoory et al., 2017) and (2) connectivity was evaluated between spatially distinct ROIs defined on the cortical template rather than between immediately adjacent electrodes, a strategy shown to improve the functional specificity of source-level connectivity estimates (Schoffelen and Gross, 2009). Reported connectivity differences should therefore be interpreted as putative inter-regional coupling patterns within this methodological framework.

#### 2.5.5 Feature extraction for classification

Feature extraction was performed on sensor-level EEG epochs ranging from −0.4 to 1.6 s relative to segment onset, targeting classification of the four experimental conditions (MD-Easy, MD-Hard, AD-replay-Easy, AD-replay-Hard). The preprocessing pipeline included notch filtering (50 and 100 Hz), band-pass filtering (2–36 Hz), baseline correction (−0.2 to 0 s), and z-score normalization across channels and participants (Bigdely-Shamlo et al., 2015; Luck, 2014).

Two types of wavelet-based features were derived. Wavelet-derived spectral features were obtained using discrete wavelet transform (DWT) with the Daubechies 4 (“db4”) wavelet (Daubechies, 1992), decomposing each EEG channel into five sub-bands. Within each sub-band, statistical measures–including mean amplitude, energy, and wavelet entropy (Rosso et al., 2001) –were calculated to characterize signal properties. Wavelet coherence features were estimated across 11 anatomically informed electrode pairs (e.g., frontal-parietal, frontal-central, interhemispheric), using Pearson correlation of wavelet coefficients to reflect functional connectivity (Grinsted et al., 2004; Sauseng and Klimesch, 2008; Torrence and Compo, 1998). All spectral and coherence features were concatenated into a unified feature vector for each epoch and subsequently entered the classification framework (Section “2.5.5 Feature extraction for classification”).

#### 2.5.6 Classification analysis

A supervised machine learning pipeline was developed to classify EEG epochs into four experimental conditions (MD-Easy, MD-Hard, AD-replay-Easy, AD-replay-Hard), utilizing a Random Forest classifier implemented in Python with the Scikit-learn library (Pedregosa et al., 2018). Feature selection within each training fold followed a two-stage procedure. Initially, a model-based ranking identified a broad set of relevant features based on their importance scores. This subset was then refined using univariate ANOVA F-statistics, yielding a final set of discriminative features.

Model performance and generalizability were evaluated using two validation schemes. In the split-sample approach, class-balanced data were stratified into training, validation, and test sets. In the Leave-One-Subject-Out (LOSO) cross-validation, each participant was iteratively held out as the test subject, with the remaining data used for training and feature selection. Class balancing was applied within each fold to mitigate bias.

Performance metrics included accuracy, macro-averaged F1 score, precision, recall, and normalized confusion matrices to illustrate condition-specific classification patterns. Feature importance scores were extracted from the trained Random Forest models–either from the full split-sample model or averaged across LOSO folds–to identify EEG features most predictive of task condition.

### 2.6 Statistical analysis

Statistical analyses were performed to examine the effects of driving mode and task complexity on EEG-derived measures, including ROI activity, frequency-domain metrics (band power and ERD/ERS), and classification features. All procedures were implemented in Python using libraries such as SciPy (Virtanen et al., 2020), MNE-Python, and PyWavelets. The significance threshold was set at α = 0.05, with Bonferroni or false discovery rate (FDR) correction applied where applicable.

Region of interest and frequency-domain effects were assessed using one-sample *t*-tests against zero within the 0.1–0.8 s post-segment window. One-tailed tests were employed for directional hypotheses and paired-sample *t*-tests were used to evaluate interaction effects, such as differences in task complexity modulation between driving modes. Effect sizes were quantified using Cohen’s d.

To address the multiplicity of tests, analyses were grouped into four families: (i) whole-brain source maps, (ii) ROI activation, (iii) frequency-domain modulation, and (iv) functional connectivity. Within each family, *p*-values were adjusted using the Benjamini–Hochberg FDR procedure. Confirmatory contrasts were tested one-tailed in line with *a priori* predictions, whereas exploratory contrasts were evaluated two-tailed. Results that did not survive FDR correction are reported descriptively as “trends.” Bootstrap resampling (5,000 iterations) was additionally applied to estimate confidence intervals and corrected *p*-values, with full results provided in Supplementary Table 3.

Wavelet-derived features (Section “2.5.4 Functional connectivity analysis”) were statistically compared across experimental conditions using paired t-tests for predefined condition pairs (e.g., MD-Easy vs. AD-replay-Easy). To evaluate global effects across EEG channels within each frequency band, *p*-values were combined using Fisher’s method.

Classifier performance metrics (accuracy, macro-averaged F1, precision, recall) obtained from split-sample and LOSO validation were tested against the theoretical chance level (25%) using binomial or permutation tests, depending on the analysis context.

Visualization of statistical results included topographic maps (via MNE-Python) to display spatial distributions of significant effects, as well as bar plots and heatmaps to summarize differences across frequency bands, channels, and ROIs. All visualizations followed established EEG reporting practices (Klimesch, 1999; Pfurtscheller and Lopes da Silva, 1999). Custom Python scripts were used for all statistical and visualization procedures.

## 3 Results

### 3.1 Effects of driving mode (Hypothesis 1)

#### 3.1.1 Source-level contrasts between active control and passive observation

To investigate how driving mode modulates large-scale brain dynamics, we computed cortical activation difference maps (MD minus AD-replay) under both easy and hard road conditions. These group-averaged maps revealed distinct spatial activation patterns shaped by the control mode, even when external stimuli and road complexity were held constant. Although no vertices survived correction for multiple comparisons, the group-level activation maps (Figure 2a, top row = Easy, bottom row = Hard) exhibited spatially coherent trends consistent with our theoretical predictions and effect size analyses. Notably, average activation differences showed moderate effect sizes (*d* ≈ 0.27–0.28) and strong within-subject consistency across conditions, justifying the inclusion of group-level visualizations as representative of mode-dependent cortical dynamics.

Six key functional ROIs involved in attentional control, sensorimotor processing, and executive function were visualized. The boundaries of these ROIs were based on standard anatomical landmarks and functional localization schemes (Tzourio-Mazoyer et al., 2002).

Specifically, under the easy condition, manual driving (MD) elicited stronger activation in the frontal and occipital cortices, reflecting increased demands on cognitive control and visual attention. In contrast, under the hard condition, AD-replay induced stronger activation in occipital and parietal regions, suggesting enhanced visual monitoring and spatial processing in the absence of active vehicle control.

To validate the feasibility of the source-space comparison approach, we also visualized the same contrasts at the individual subject level. In a representative participant (SUB_2), as shown in Figure 2b (individual subject maps; top row = Easy, bottom row = Hard, lateral and medial views), similar activation differences were observed: mPFC and occipital increases during MD under easy conditions, and greater PPC and medial motor cortex activation during AD-replay under hard conditions. The observed consistency suggests that group-level effects were not artifacts of averaging but reflect underlying individual trends. This confirms that the group-level findings are not driven by a small subset of participants and that the analysis pipeline is appropriate for revealing condition-specific differences.

These source-space patterns support Hypothesis 1 by showing that neural activity differs substantially between manual and automated driving, even when participants are exposed to identical external stimuli and road complexity. The medial prefrontal cortex (mPFC) was more active during MD, consistent with higher demands on executive control and sustained attention. The elevated PPC and occipital activation during AD-replay suggests a shift toward passive visual monitoring and spatial estimation when participants were not actively engaged in control.

As a complementary validation, we trained a binary classifier on MD epochs and tested it on AD-replay. The model failed to generalize, achieving 58.4% accuracy–a marginal gain over the 50% chance level that was not statistically reliable (permutation/binomial test, n.s.). This failure supports the conclusion that mode-specific neural differences hinder cross-mode transfer. Consistent with this, we observed high within-subject reliability (*r* = 0.88 ± 0.065) but substantial between-subject variability (CV > 17%), which can also obscure group-level effects.

Together, these source-level findings, supported by both group-level and individual data, robustly validate Hypothesis 1 and establish that manual and automated driving modes elicit distinct and spatially distributed patterns of neural activity. Driving mode modulates brain engagement even when external conditions are held constant. Manual driving elicited stronger engagement of mPFC and visual cortices, consistent with increased cognitive demand, while automated driving was characterized by elevated activity in PPC and medial motor areas, indicating a shift toward passive sensory-motor monitoring. It should be noted that these anatomical attributions are treated as putative given the 29-channel template-based source model; importantly, converging sensor-space topographies (Supplementary Figure 3, Sensor-space topographies for all contrasts.) exhibited consistent frontal/occipital increases during MD and posterior/parietal increases during AD, corroborating the source-level contrasts.

#### 3.1.2 ROI activation differences

**FIGURE 3:**
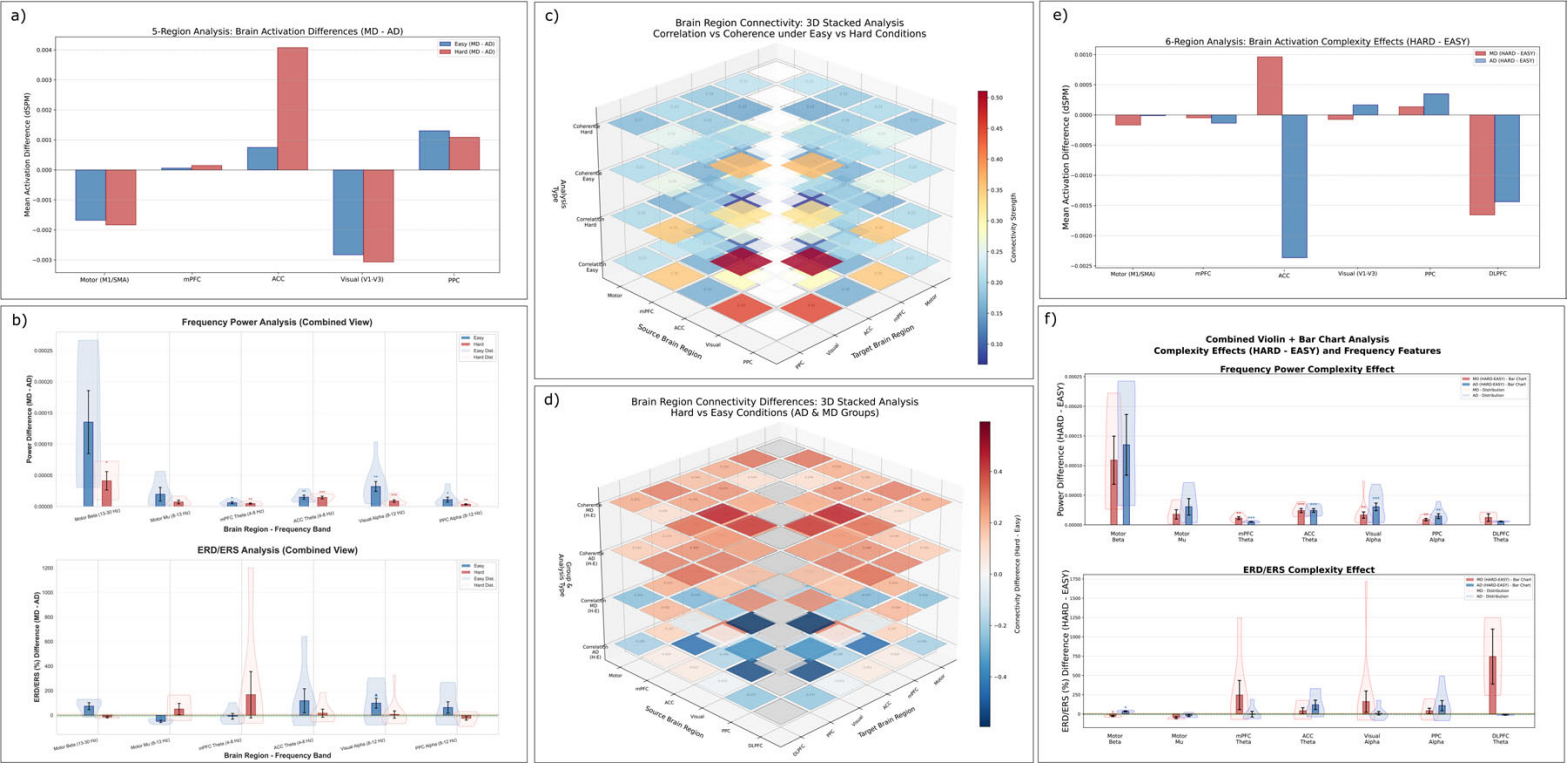
Neural activation and functional connectivity differences across driving modes and task complexity levels. **(a)** Mean activation differences (MD minus AD-replay) across five functionally defined brain regions under easy and hard conditions, computed from source-localized dSPM values. **(b)** Frequency-domain power (top) and ERD/ERS (bottom) comparisons across selected ROIs and frequency bands. Bar and violin plots show MD minus AD-replay differences across easy and hard conditions. **(c)** 3D stacked heatmap of ROI-to-ROI functional connectivity (coherence and correlation) across manual vs. automated driving (MD/AD-replay) and easy vs. hard task conditions. Each layer represents one connectivity type under a specific condition. **(d)** Layered 3D matrix showing connectivity differences between hard and easy conditions (HARD - EASY) for both MD and AD-replay groups, revealing task complexity modulation of large-scale brain networks. **(e)** Complexity-induced activation changes (HARD - EASY) for MD and AD-replay separately, showing region-specific effects of task demand on cortical engagement. **(f)** Frequency power (top) and ERD/ERS (bottom) differences between hard and easy conditions across selected ROIs, separately for MD and AD-replay. Combined bar and violin plots illustrate both magnitude and inter-subject variability. Error bars represent standard error of the mean (SEM); asterisks indicate statistically significant differences (*p* < 0.05). ROIs include the medial motor area (M1/SMA), medial prefrontal cortex (mPFC), anterior cingulate cortex (ACC), visual cortex (V1–V3), posterior parietal cortex (PPC), and dorsolateral prefrontal cortex (DLPFC). All activation and frequency power values are reported in arbitrary units (a.u., z-scored SNR or normalized power), ERD/ERS values are expressed as % change from baseline, and connectivity measures represent dimensionless correlation or coherence coefficients.

To further examine how driving mode influences localized cortical engagement, region-of-interest (ROI) analyses were conducted across five functionally relevant brain areas: the medial motor areas (M1 and SMA), medial prefrontal cortex (mPFC), anterior cingulate cortex (ACC), visual cortex (V1–V3), and posterior parietal cortex (PPC). These ROIs were selected based on established functional relevance and are illustrated in Figure 2c (ROI schematic of left lateral and medial views). Distinct and consistent mode-dependent differences in neural activity were observed across these ROIs (Figure 3a, ROI bar plots, MD − AD contrasts under easy and hard conditions). Detailed statistical outcomes, including original tests and bootstrap resampling with FDR correction, are reported in Supplementary Table 3.

The medial motor areas exhibited greater activation during AD-replay compared to MD, reflected in negative differences (MD − AD-replay < 0) for both easy (*M* = −0.0017) and hard conditions (*M* = −0.0018). While not reaching statistical significance, this consistent negative trend likely indicates compensatory motor prediction or anticipatory processes associated with passive driving control, alongside possible muscle-related activations (Pfurtscheller and Lopes da Silva, 1999; Pfurtscheller and Neuper, 1997). Consistent with this interpretation, recent studies have shown that motor regions can display anticipatory activity even without overt movement, including mu suppression during passive action observation (Krol and Jellema, 2022; Syrov et al., 2023), as well as predictive EEG components preceding steering actions in driving simulations (Di Liberto et al., 2021). Bootstrap resampling (5,000 iterations) further indicated uncorrected effects in the AD > MD direction for both Easy (*p* = 0.0164, 95% CI [−0.0016, −0.0001]) and Hard (*p* = 0.018, 95% CI [−0.0016, −0.0002]) conditions (see Supplementary Table 3). However, because Motor ROI activation analyses were exploratory within family 2, these effects did not survive FDR correction and are therefore reported as trends.

In contrast, the mPFC showed an opposite trend, displaying increased activation during manual driving (MD − AD-replay > 0), with modest statistical significance particularly evident under hard conditions (e.g., right hemisphere rostral middle frontal ROI: *M* = 0.0065, *p* < 0.001; bootstrap FDR-adjusted *p* = 0.77, not significant after correction, descriptive only). Given the established role of mPFC in executive control and sustained attention (Miller and Cohen, 2001), this finding supports the interpretation that active vehicle control demands greater cognitive effort under challenging conditions.

The ACC also exhibited greater activation during MD compared to AD-replay, especially under high task complexity (Hard condition mean: 0.0041). Notably, caudal anterior cingulate regions showed significant differences in favor of manual driving under both conditions, particularly pronounced in the hard condition (left hemisphere ROI: *M* = 0.0120, *p* < 0.001; right hemisphere ROI: *M* = 0.0083, *p* < 0.001). This aligns well with the ACC’s known involvement in cognitive control and attentional resource allocation, further highlighting the cognitive demands associated with manual driving under heightened task complexity.

The visual cortex demonstrated greater activation during automated driving (negative MD − AD-replay differences), suggesting a shift toward enhanced visual monitoring when active manual engagement is absent (Corbetta and Shulman, 2002). Detailed analyses confirmed this effect, as evidenced by consistent negative differences across multiple visual cortex ROIs, particularly within fusiform regions (left hemisphere, hard: *M* = −0.0098; uncorrected *p* < 0.05, but not significant on the one-sided test because the effect was opposite to the *a priori* direction) and lingual regions.

In the PPC, increased activation during manual driving relative to automated driving was observed, particularly notable in the inferior parietal regions bilaterally (left hemisphere inferior parietal: hard *M* = 0.0035, *p* < 0.001; easy *M* = 0.0036, *p* = 0.001; right hemisphere inferior parietal: hard *M* = 0.0035, *p* < 0.001; easy *M* = 0.0035, *p* < 0.001). Given the PPC’s established role in spatial attention and sensorimotor integration, this finding is consistent with increased cognitive and attentional demands associated with manual control. Although superior parietal and supramarginal regions demonstrated variability and mostly non-significant differences, the robust findings in inferior parietal areas strongly support enhanced spatial attentional processes during manual driving. These differences did not remain significant after FDR correction and are reported descriptively.

Statistical tests largely supported these observations. Despite certain individual ROI results showing variability, the overall pattern clearly favored increased cognitive and attentional processing during manual driving in mPFC, ACC, and PPC regions. These ROI-level findings collectively reinforce Hypothesis 1, highlighting distinct regional neural activation profiles elicited by manual versus automated driving, with manual control preferentially engaging cognitive and attentional networks, and automated driving prompting compensatory motor and visual monitoring processes.

#### 3.1.3 Frequency-domain modulation

To investigate how driving mode influences frequency-specific neural dynamics, we conducted analyses of frequency band power and event-related desynchronization/synchronization (ERD/ERS) across five functionally relevant brain regions: medial motor areas (Motor), medial prefrontal cortex (mPFC), anterior cingulate cortex (ACC), visual cortex (Visual), and posterior parietal cortex (PPC). These analyses targeted physiologically meaningful frequency bands, specifically beta (13–30 Hz) and mu (8–13 Hz) for motor areas, theta (4–8 Hz) for frontal midline regions (mPFC and ACC), and alpha (8–12 Hz) for visual and parietal cortices The corresponding results are shown in Figure 3b (top row: power spectra across ROIs; bottom row: ERD/ERS violin and bar plots).

The analysis revealed significant modulation of neural spectral activity by driving mode, differing distinctly across brain regions. In frontal midline regions, theta-band power significantly increased during manual driving (MD) relative to Automated Driving - Passive Replay (AD-replay), particularly within the mPFC (easy: *p* = 0.011; hard: *p* = 0.004) and ACC (easy: *p* = 0.004; hard: *p* < 0.001). Bootstrap resampling (5,000 iterations) confirmed these effects with FDR-adjusted *p* < 0.001 across all conditions, indicating highly robust increases in frontal theta power during manual control. This elevated theta activity likely reflects enhanced cognitive control, which supports conflict monitoring and executive regulation (Cavanagh and Frank, 2014; Eisma et al., 2021), together with sustained attention demands required to maintain task engagement (McFerren et al., 2021) during manual control under both easy and complex driving conditions.

In the visual cortex, alpha-band power during manual driving (MD) significantly exceeded that of automated driving–passive replay (AD-replay) for both easy (*p* = 0.001) and hard (*p* < 0.001) conditions. This was further evidenced by significant alpha synchronization (ERS: +97.9%, *p* = 0.039) in the MD-easy condition relative to baseline. Bootstrap analysis confirmed consistent uncorrected trends (*p* < 0.001) in the MD > AD direction, but as visual alpha was treated as exploratory, these results did not undergo FDR correction and are reported trend. A similar MD-related increase in alpha power was observed in the PPC (easy: *p* = 0.018; hard: *p* = 0.004). Bootstrap resampling also showed consistent uncorrected trends (*p* < 0.001) but as exploratory findings they are presented descriptively. Because visual input was held constant across modes, these alpha effects likely reflect mode-dependent top-down modulation rather than differences in sensory stimulation.

Motor areas showed significant beta-band power increases during manual driving under hard conditions (*p* = 0.047), suggesting heightened motor preparation during active driving while bootstrap resampling confirmed this effect as highly robust (FDR-adjusted *p* < 0.001). Moreover, mu-band ERD analyses revealed significant desynchronization under easy conditions (−50.11%, *p* = 0.019), which also remained significant after bootstrap correction (FDR-adjusted *p* < 0.001), confirming robust sensorimotor integration during manual control. further supporting enhanced sensorimotor integration during manual control (Pfurtscheller and Lopes da Silva, 1999; Pfurtscheller and Neuper, 1997). This interpretation aligns with recent findings showing that beta oscillations are now recognized as key contributors to motor prediction and the stabilization of sensorimotor states (Barone and Rossiter, 2021), while the phase dynamics of mu and beta rhythms have been directly linked to corticospinal excitability and integrative motor processes (Wischnewski et al., 2022). In naturalistic driving contexts, predictive EEG components preceding steering actions further demonstrate that both μ and β rhythms support anticipatory and integrative mechanisms that are strengthened during manual control (Di Liberto et al., 2021). This pattern highlights that sensorimotor integration–linking visual inputs with motor planning–is a key neural substrate of active driving engagement, and its attenuation under AD-replay reflects the reduced coupling between perception and action in passive observation states.

Collectively, these frequency-domain results align closely with findings from source-space and ROI-level activation analyses, collectively supporting the interpretation that manual driving uniquely engages cognitive control mechanisms (theta-band in mPFC and ACC), intensified visual attention (visual cortex alpha synchronization), and motor preparation processes (motor cortex beta modulation), underscoring distinct neural dynamics between manual and automated driving modes.

#### 3.1.4 Functional connectivity patterns between brain regions

Pairwise functional connectivity between selected brain regions was analyzed using correlation and coherence measures to further elucidate how driving mode modulates large-scale neural coordination. Functional connectivity heatmaps (Figure 3c, 3D-stacked matrices; bottom two layers = correlation-based connectivity under Easy and Hard, top two layers = coherence-based connectivity under Easy and Hard) were generated to illustrate inter-regional coupling patterns across motor, medial prefrontal cortex (mPFC), anterior cingulate cortex (ACC), visual cortex, and posterior parietal cortex (PPC) under both easy and hard driving conditions.

The correlation-based analysis highlighted distinct driving mode-dependent connectivity profiles. During manual driving, the motor and visual cortices displayed moderate positive correlations under easy conditions (*r* = 0.220), reflecting integrated sensorimotor processing during relatively low-demand driving scenarios. Additionally, connectivity between the PPC and mPFC was notably stronger (*r* = 0.354 under easy; *r* = 0.355 under hard conditions), suggesting enhanced coordination between spatial attention and executive control regions during active driving.

Interestingly, motor-mPFC connectivity revealed a moderate positive correlation that strengthened under easy conditions (*r* = 0.340), potentially indicative of a synchronized integration between motor control and executive functions when attentional resources are readily available. Conversely, correlations between the ACC and other regions remained low across both complexity conditions (e.g., motor-ACC under hard conditions, *r* = 0.067), implying that ACC activation during driving tasks might function somewhat independently from these other networks, or reflect highly state-specific engagements.

The coherence analysis, complementary to the correlation-based assessment, further validated these observations. Functional coherence between visual and parietal regions was moderately robust under both easy (coherence = 0.327) and hard conditions (coherence = 0.365), reflecting stable visual-spatial processing coordination regardless of task complexity. Connectivity between motor and mPFC areas demonstrated lower coherence (e.g., hard condition coherence = 0.215), suggesting limited direct frequency-based interaction between motor execution and higher cognitive processes during heightened task demands.

In conclusion, these functional connectivity patterns reinforce Hypothesis 1, demonstrating clear neural differentiation between manual and automated driving modes, not only in individual region activation intensities but also in their inter-regional communication patterns. Manual driving elicited more integrated sensorimotor and cognitive coordination, particularly evident between motor and frontal regions under low task complexity, and robust parietal-prefrontal connectivity across complexity levels. Conversely, automated driving appeared associated with reduced fronto-motor integration and comparatively stable visual-spatial network interactions, supporting a shift toward passive visual monitoring, and decreased cognitive-motor integration. These mode-specific connectivity distinctions highlight the profound impact driving mode exerts on large-scale brain network organization, independent of external road condition demands.

Together, the results from source-level mapping, ROI analyses, spectral dynamics, functional connectivity, and cross-condition classification consistently support Hypothesis 1: manual and automated driving engage the brain in fundamentally distinct ways. Manual driving elicited increased activation in frontal and parietal regions, elevated theta and alpha synchronization, and tighter fronto-motor connectivity, reflecting heightened demands on executive control, visual processing, and spatial attention. In contrast, automated driving was characterized by enhanced occipital activation and weakened inter-regional coupling, suggesting a shift toward passive visual monitoring with reduced cognitive engagement. These mode-specific differences were consistently observed across matched road conditions, indicating that control state alone robustly shapes neural dynamics. I next examined whether task complexity (easy vs. hard) further modulates these patterns, and whether such effects interact with the driving mode.

### 3.2 Effects of task complexity and interaction with mode (Hypothesis 2)

#### 3.2.1 Source-level contrasts of hard vs. easy

**FIGURE 4:**
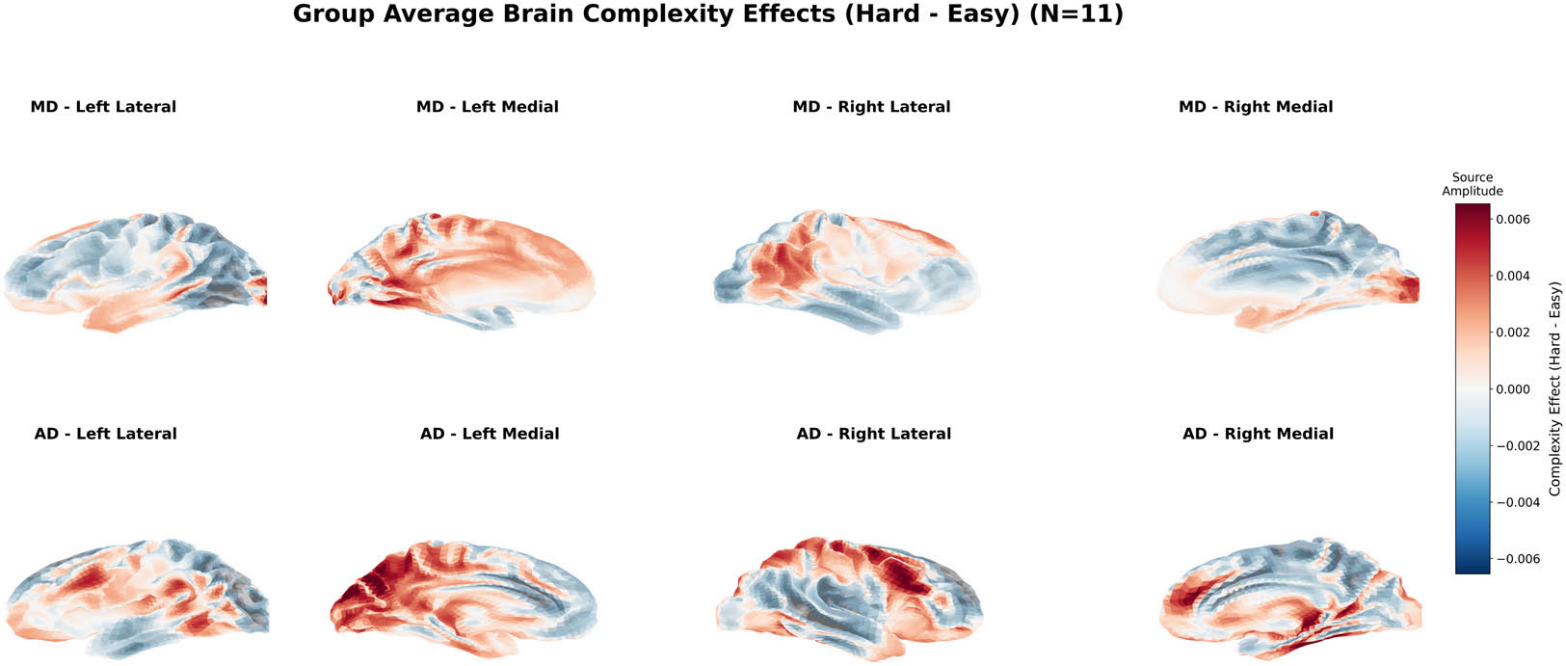
Group average brain complexity effects. Group-level cortical activation contrasts between Hard and Easy segments (Hard - Easy) under different driving modes (*N* = 11). Surface maps illustrate average source-level activity differences for manual driving (MD, top row) and automated driving (AD, bottom row), rendered from four standard FreeSurfer views (left lateral, left medial, right lateral, right medial). Warmer colors indicate increased activation during hard segments, while cooler colors denote decreased activation. Under MD, focal increases were observed in medial frontal, occipital, and parietal cortices, including the supplementary motor area (SMA), anterior cingulate cortex (ACC), and posterior parietal cortex (PPC), reflecting elevated cognitive and visuospatial processing demands. In contrast, AD produced more diffuse and widespread activation across posterior cortices, particularly in the occipital and temporal–parietal regions, suggesting less selective cortical engagement during increased complexity.

Cortical activation difference maps (Hard − Easy) separately for manual driving (MD) and Automated Driving - Passive Replay (AD-replay) were computed in this part to investigate how task complexity modulates cortical activation. These maps revealed distinct spatial patterns that varied depending on the control mode, as illustrated in Figure 4 (top row: MD, bottom row: AD; four cortical views each).

Under MD, increased task complexity led to localized activation increases in the medial frontal cortex, notably within the supplementary motor area (SMA) and anterior cingulate cortex (ACC), as well as in parietal and occipital regions. These areas are broadly associated with cognitive control, error monitoring, and visuospatial integration (Pfurtscheller and Neuper, 1997). The focal recruitment of frontal and parietal regions is consistent with their established roles in maintaining executive function and attentional allocation during demanding tasks (Wascher et al., 2018).

Automated Driving - Passive Replay, by contrast, exhibited more spatially diffuse activation increases under hard conditions, with prominent changes observed in the occipital cortex and temporal–parietal junction. The absence of frontal specificity suggests a less efficient, potentially compensatory, pattern of neural engagement. This broader activation profile aligns with findings frequently observed during passive monitoring or reduced cognitive involvement when control is delegated to automation (McDonnell et al., 2023; McWilliams and Ward, 2021; Merat et al., 2014; Parasuraman and Manzey, 2010).

Although both conditions exhibited elevated cortical activity in response to increasing complexity, their topographical profiles differed. MD elicited more anatomically selective activation in task-relevant areas, whereas AD-replay was associated with widespread but less targeted cortical changes. This suggests that effective cortical switching between task-relevant and diffuse networks may be contingent on the level of volitional engagement: when drivers are actively in control, adaptive recruitment is localized to frontal and parietal circuits that support conflict monitoring and attentional allocation (Botvinick et al., 2001; Wascher et al., 2018), whereas passive observation under automation elicits a more diffuse, less efficient activation profile consistent with reduced cognitive involvement (McDonnell et al., 2023; McWilliams and Ward, 2021; Merat et al., 2014). Such switching is consistent with broader accounts of adaptive cortical network dynamics (Aston-Jones and Cohen, 2005; Raichle, 2015), which posit that task-related reorganization is contingent on motivational state and active engagement. Again, these regional inferences are reported as putative, but the observed Hard–Easy topographies at the sensor level (Supplementary Figure 3) were spatially consistent with the source-localized effects, reinforcing the robustness of the observed mode × complexity differences.

Overall, these results support Hypothesis 2 by demonstrating that task complexity interacts with control mode to shape neural engagement in terms of both magnitude and spatial specificity.

#### 3.2.2 ROI-specific complexity effects and mode × complexity interaction

We next examined activation differences (Hard − Easy) across six predefined ROIs under both MD and AD-replay, as shown in Figure 3e (bar plots of Hard–Easy contrasts; red = MD, blue = AD across Motor, mPFC, ACC, Visual, PPC, and DLPFC) to further explore the modulatory effect of task complexity. The pattern of results varied notably by both brain region and control mode.

In the dorsolateral prefrontal cortex (DLPFC), task complexity elicited reduced activation in both MD and AD-replay, with the suppression more pronounced in the automated mode. This pattern may suggest a downregulation of working memory or executive switching demands when task control is offloaded. A similar mode-specific divergence emerged in the anterior cingulate cortex (ACC): while MD showed a slight increase in activation, consistent with demand monitoring, AD-replay instead exhibited a robust decrease. Such reductions may reflect diminished engagement of conflict monitoring systems in passive control states.

The posterior parietal cortex (PPC) displayed weak and divergent responses across modes, with marginal increases under AD-replay and nearly flat differences under MD. This finding contrasts with prior expectations of enhanced spatial attention under high complexity, suggesting that PPC engagement may depend more on task type than on visual scene complexity alone.

In the visual cortex, task complexity led to slight activation increases under MD, aligning with increased visual vigilance. However, the opposite trend was observed under AD-replay, where activity decreased, potentially indicating a reduced need for active visual tracking during automation.

Interestingly, the medial prefrontal cortex (mPFC) showed little modulation under either mode, in contrast to its typically hypothesized role in demand-dependent executive control. The motor cortex (M1/SMA) also exhibited small decreases across both MD and AD-replay, suggesting that increased complexity might not translate to heightened motor planning in the absence of explicit motoric challenge.

Together, these ROI-level findings illustrate that brain responses to complexity are highly contingent on the mode of control. Manual driving recruits frontal and posterior regions in ways consistent with adaptive increases in cognitive and visual engagement, while automation appears to either blunt or reverse these effects across multiple systems.

#### 3.2.3 Frequency-domain complexity modulation

Spectral analyses confirmed the interaction between task complexity and control mode. The results revealed that neural modulation patterns varied substantially depending on both brain region and control mode (Figure 3f, top panel: frequency power differences across ROIs; bottom panel: ERD/ERS dynamics). In the frontal midline and DLPFC, theta-band power increased significantly during hard segments under manual driving (MD), consistent with enhanced executive demand and adaptive control allocation. In contrast, no such increase was observed under AD-replay, suggesting that the cognitive control network remained largely disengaged when drivers were not actively involved in vehicle control. This differential pattern was also reflected in the ACC, where theta power was elevated in MD but less consistently modulated in AD-replay, indicating diminished recruitment of conflict monitoring processes during automation, particularly a striking decrease in activation under hard conditions in AD-replay.

In the visual cortex, alpha-band power decreased under MD-hard, consistent with intensified visual information processing under increased task load. Interestingly, the same region showed increased alpha power in AD-replay-Hard, possibly reflecting visual disengagement or a shift toward passive sensory gating under automated control. A similar pattern emerged in the posterior parietal cortex (PPC): alpha desynchronization was more pronounced under MD than AD-replay, implying that spatial attention and sensorimotor integration were selectively enhanced during active driving.

The motor cortex presented a more complex picture. Neither beta nor mu power increased with task complexity under MD; rather, slight decreases were observed. Given the concurrent elevation of prefrontal theta activity, this may reflect a strategic reallocation of cognitive resources–favoring executive control over motor readiness–as task difficulty increases. Furthermore, ERD/ERS patterns in the motor cortex were variable across individuals and showed no consistent trend, while in regions like DLPFC and ACC, ERS responses were more prominent in AD-replay than in MD, possibly indicating altered or compensatory recruitment of higher-order networks during passive monitoring.

Collectively, these frequency-domain analyses demonstrate that task complexity elicits structured, region-specific neural modulations primarily under manual control, whereas the same task demands evoke attenuated, inconsistent, or even paradoxical responses under automation. These findings reinforce the view that driver engagement acts as a critical modulator of cortical adaptation to environmental complexity, whereby active control facilitates targeted neural reorganization while passive monitoring yields broader but less effective responses. Because task difficulty was manipulated in an interleaved manner within fixed engagement modes, these effects can be interpreted as reflecting the brain’s engagement-dependent adaptation to varying environmental demands. This interpretation is further supported by recent work showing that adaptive recruitment of prefrontal and cingulate systems scales with task demand (Tang et al., 2022), that cortical responsiveness is state-dependent (Perera et al., 2024), and that anticipatory neural signatures in naturalistic driving (Di Liberto et al., 2021) as well as diminished frontal activation during automation (McDonnell et al., 2023) highlight how volitional involvement shapes the brain’s adaptive capacity. Bootstrap analyses provided strong confirmation of theta power increases in both mPFC and ACC during MD-hard segments, highlighting the robustness of frontal control network engagement under demanding conditions as reported in Supplementary Table 3.

#### 3.2.4 Functional connectivity modulation

Functional connectivity changes (Hard − Easy) across six task-relevant brain areas were evaluated using both correlation- and coherence-based metrics. The results are illustrated in Figure 3d (3D-stacked matrices; bottom two layers = correlation-based connectivity for MD and AD, top two layers = coherence-based connectivity for MD and AD). This representation delineates how task complexity influences inter-regional coordination under each control mode.

Generally, coherence-based connectivity demonstrated greater sensitivity to task complexity compared to simple correlation. Under manual driving (MD), hard segments elicited widespread increases in coherence, notably across fronto-parietal and fronto-motor pathways (e.g., mPFC–PPC, ACC–Motor, DLPFC–PPC). The most prominent enhancements were observed in mPFC–PPC (Δ coherence = +0.276) and ACC–PPC (Δ = +0.450). These patterns align with the heightened cognitive and spatial coordination demands prevalent in active control conditions.

Conversely, during AD-replay, the coherence increases were more uniformly distributed across regions, encompassing occipital–frontal and occipital–parietal pairs. While these connections also exhibited moderate enhancement (e.g., ACC–DLPFC: Δ = +0.340), the absence of clear functional clustering suggests a less targeted network adaptation. This might indicate a more passive or compensatory monitoring state when control authority is externally assigned.

Correlation-based results yielded a different picture. Under MD, several connections–particularly those involving the visual cortex (e.g., Visual–mPFC, Visual–PPC)–exhibited marked decreases, pointing toward a shift toward more decoupled processing streams under high complexity. This decoupling became even more apparent under AD-replay, where negative changes were observed between core executive and sensory nodes (e.g., mPFC–PPC: Δr = −0.460; ACC–Visual: Δr = −0.132). Such reductions in zero-lag correlations could signify diminished synchronous activation or increased variability in regional timing during automated control.

These illuminate that task complexity modulates functional integration in a mode-specific fashion. Manual driving is associated with increased coherence within executive–sensorimotor networks, supporting the engagement of dynamic, top-down regulatory loops. Conversely, connectivity changes under AD-replay appear more diffuse and less specialized, consistent with the broader pattern of attenuated or redistributed engagement observed in prior activation and frequency-based results.

Overall, findings from these 3 aspects provide strong support for Hypothesis 2: task complexity significantly modulates brain activation, and this modulation is shaped by the level of driver engagement. Under manual control, increasing difficulty enhances engagement of cognitive, visual, and spatial networks in a structured and localized manner, with robust, region-specific enhancements in neural activity. This included elevated theta power in the dorsolateral prefrontal cortex (DLPFC) and frontal midline–indicative of enhanced executive control–and pronounced alpha desynchronization in the posterior parietal cortex (PPC), reflecting heightened visuospatial attention. Mild increases in anterior cingulate cortex (ACC) activation were also observed, consistent with greater conflict monitoring demands. However, contrary to initial expectations, motor cortex activity decreased under high-complexity conditions, potentially reflecting cognitive overload or resource reallocation.

In contrast, AD-replay exhibited minimal or paradoxical changes in control-related regions. The only region exhibiting robust task-related modulation in AD-replay was the occipital cortex, suggesting either compensatory visual processing or a shift toward passive sensory monitoring. Whole-brain activation maps (Figure 4) further illustrated this pattern: MD elicited spatially focused activation in frontal and parietal regions, while AD produced widespread, less localized responses concentrated in posterior cortices.

Taken together, these results validate Hypothesis 2 and reinforce Hypothesis 1 by demonstrating that not only do MD and AD-replay differ in baseline neural activity, but they also exhibit fundamentally different neural adaptations to increasing task demands. Active control facilitates structured, functionally aligned cortical modulation in response to complexity, whereas passive driving induces attenuated or diffuse responses, underscoring the role of volitional engagement in shaping brain dynamics during complex tasks.

### 3.3 Classification analysis

#### 3.3.1 Classification performance and confusion matrix

**FIGURE 5:**
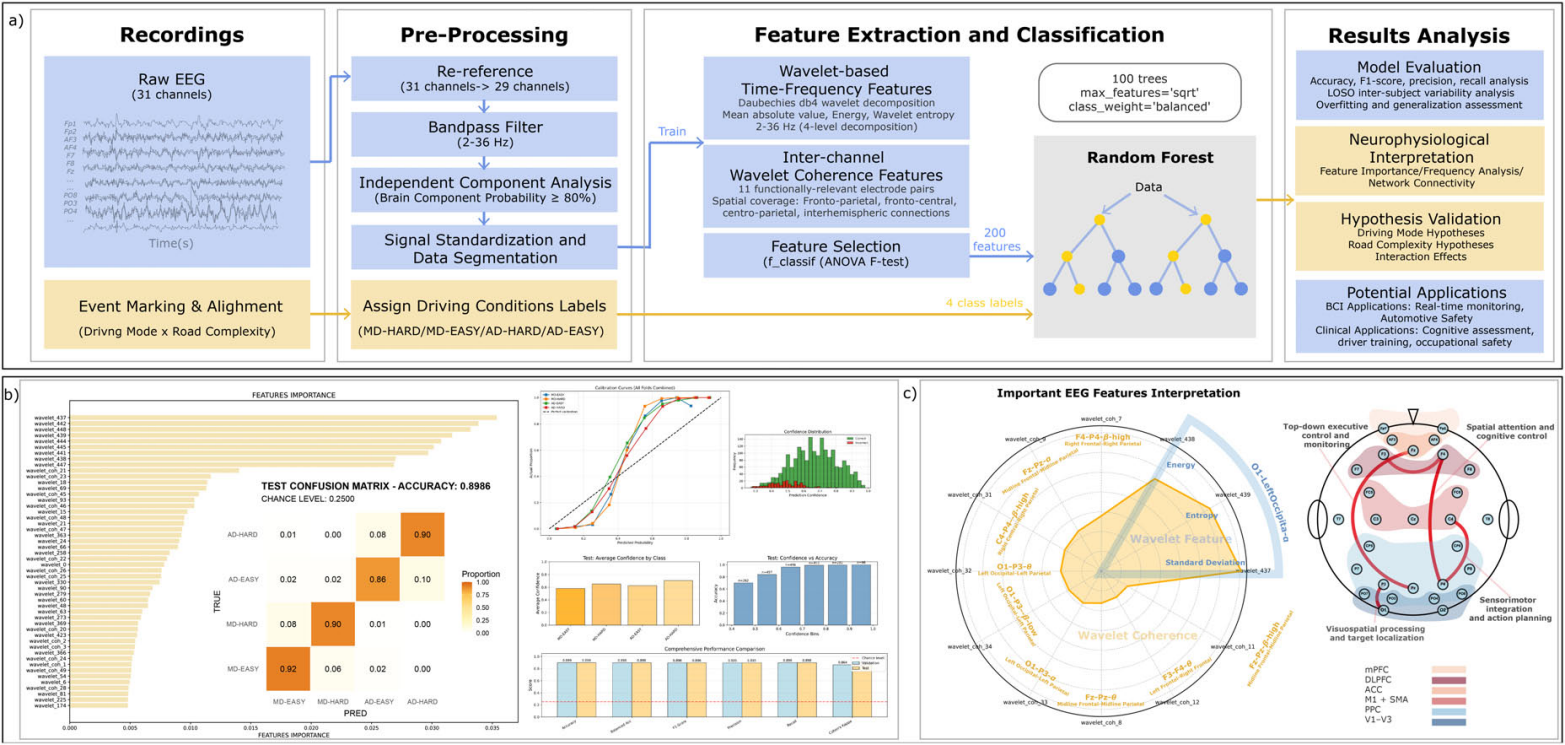
Electroencephalography-based classification pipeline and feature analysis results. **(a)** Overview of the classification pipeline. Raw EEG signals from 31 channels were preprocessed via re-referencing, bandpass filtering (2–36 Hz), and independent component analysis (ICA) for artifact removal. Driving condition labels (MD-HARD, MD-EASY, AD-REPLAY-HARD, AD-REPLAY-EASY) were assigned based on event markers. Feature extraction included wavelet-based time-frequency features and inter-channel wavelet coherence metrics across spatial and spectral dimensions. Feature selection was performed using F-statistics (ANOVA), and a Random Forest classifier was trained to distinguish between four conditions. **(b)** The classification outcomes and key discriminative features. All transformations that rely on dataset statistics–z-scoring (applied to wavelet-power and wavelet-coherence features) and ANOVA-F feature selection–were fit only within the training fold (nested preprocessing). Cross-validation used 5-fold GroupKFold with segment/lap-level grouping, ensuring temporally adjacent epochs from the same sequence were never split across folds. Shown are ranked feature importance (left), the held-out confusion matrix (center; accuracy = 0.8986 ± 0.0204, macro-F1 = 0.8956, chance = 0.25), and summary reliability/calibration (right), confirming the model’s robustness and interpretability. **(c)** Interpretation of key EEG features contributing to driving condition classification. The left radar plot highlights top-ranking EEG features from the classification model, grouped by feature type (wavelet power and wavelet coherence), frequency band, and involved electrode pairs. These include occipital alpha power, frontal theta, and fronto-parietal beta coherence, which align with prior ROI-based and frequency-domain findings. The right schematic maps these features onto corresponding brain regions, illustrating their involvement in functional networks related to executive control (mPFC, DLPFC), sensorimotor planning (M1/SMA), visuospatial integration (PPC), and visual processing (V1–V3). Together, this visualization links model-derived features to established neurocognitive substrates of manual and automated driving.

To rigorously test whether EEG features could reliably differentiate the cognitive states induced by varying driving modes and road complexities, we trained a four-class Random Forest classifier. This within-subject analysis is intended to identify discriminative EEG features and assess their neurophysiological plausibility under a leakage-safe evaluation, rather than to build a cross-subject model; cross-subject generalization is evaluated separately in Section “3.3.3 Generalization across subjects (LOSO performance).” This model leveraged a rich set of wavelet-based EEG spectral and coherence features (Figure 5a, feature extraction and classification pipeline) to discriminate between the MD-Easy, MD-Hard, AD-replay-Easy, and AD-replay-Hard conditions. To preclude information leakage, all transformations that rely on dataset statistics (z-scoring for wavelet-power and wavelet-coherence features and ANOVA-F feature selection) were fit only within the training fold (nested preprocessing), and cross-validation used a 5-fold GroupKFold with segment/lap-level grouping; in addition, epochs temporally adjacent to the held-out segments were excluded from the training portion within each fold (a purged-gap scheme). The classifier demonstrated remarkable predictive power, achieving a cross-validated test accuracy of 89.86% ± 2.04%, significantly outperforming the 25% chance level. This performance was mirrored by a macro-averaged F1 score of 0.8956 (Figure 5b, right panels, model evaluation metrics). Per-fold confusion matrices and calibration curves are provided in Supplementary Figure 2.

A closer inspection of the confusion matrix (Figure 5b, center panel) reveals a highly accurate classification profile across conditions. Notably, both manual driving states showed strong discriminability, with MD-Hard achieving a recall of 90.5% and precision of 93.5%, and MD-Easy attaining a recall of 92.2% and precision of 86.5%. The AD-replay-Hard condition was also classified with high fidelity (recall = 90.3%, precision = 91.5%), while AD-replay-Easy exhibited slightly lower but still robust performance (recall = 86.1%, precision = 86.5%). These patterns are consistent with the updated leakage-safe evaluation, with fold-wise results detailed in the Supplementary Figure 2. The classifier’s high overall performance, supported by confidence-level analyses and balanced metrics, underscores the distinct neural signatures elicited by manual versus automated control under varying task complexities.

To further ensure that classification performance was not driven by residual ocular or muscle artifacts, we quantified ICA/ICLabel component classifications across conditions and tested robustness to sensor-space exclusions and stricter thresholds. Supplementary Table 1 summarizes the distribution of artifactual components (eye, muscle, cardiac, line noise, other) across MD and AD-replay, revealing no significant differences and in fact numerically fewer artifacts in MD. Supplementary Table 2 reports classifier accuracies when excluding peripheral (ocular-prone) and central motor channels, as well as when varying ICLabel thresholds from 0.5 to 0.9. In all cases, classification performance remained substantially above chance and comparable to the full-sensor 0.8-threshold pipeline, demonstrating that results are not dependent on artifact-prone components or channels.

#### 3.3.2 Feature importance and neurophysiological correlates

An analysis of the features driving the classifier’s performance provided powerful neurophysiological validation for our earlier findings (Figure 5b, left panel: feature importance ranking; Figure 5c, radar plot of feature groups and cortical schematic of contributing regions). At the local level, alpha-band power from the occipital cortex (electrodes O1 and O2) and theta-band power from the frontal midline (AF3, Fz, AF4) and dorsolateral prefrontal cortex (DLPFC) regions (F3, F4) emerged as highly influential. This highlights a key dynamic: the brain’s regulation of visual processing via occipital alpha–characterized by suppressed activity during MD conditions and elevated levels during AD-replay-Hard scenarios–and its management of executive control via frontal theta serve as a primary basis for distinguishing between the passive monitoring of automated driving and the active engagement of manual control.

Beyond local activity, the model drew heavily on network-level coherence metrics, underscoring that interregional communication is crucial for differentiating these cognitive states. Specifically, wavelet coherence within the theta and beta bands, predominantly linking frontal and parietal electrodes (e.g., Fz–Pz, F4–P4, C4–P4), were among the most discriminative features. This finding demonstrates that the synchronized activity within the fronto-parietal network, a substrate for top-down executive control, spatial attention, visuomotor integration, and sensorimotor coordination, forms a robust signature of the heightened cognitive demands inherent to manual driving.

In essence, the features that enabled successful classification were not random but were the very same neurophysiological markers identified in our preceding analyses. This powerful convergence between traditional statistical validation and a predictive machine learning framework offers a holistic confirmation: the differences between driving modes are not just statistically significant but are robust and patterned enough to allow for accurate, single-trial-level prediction of a driver’s cognitive state.

#### 3.3.3 Generalization across subjects (LOSO performance)

**FIGURE 6:**
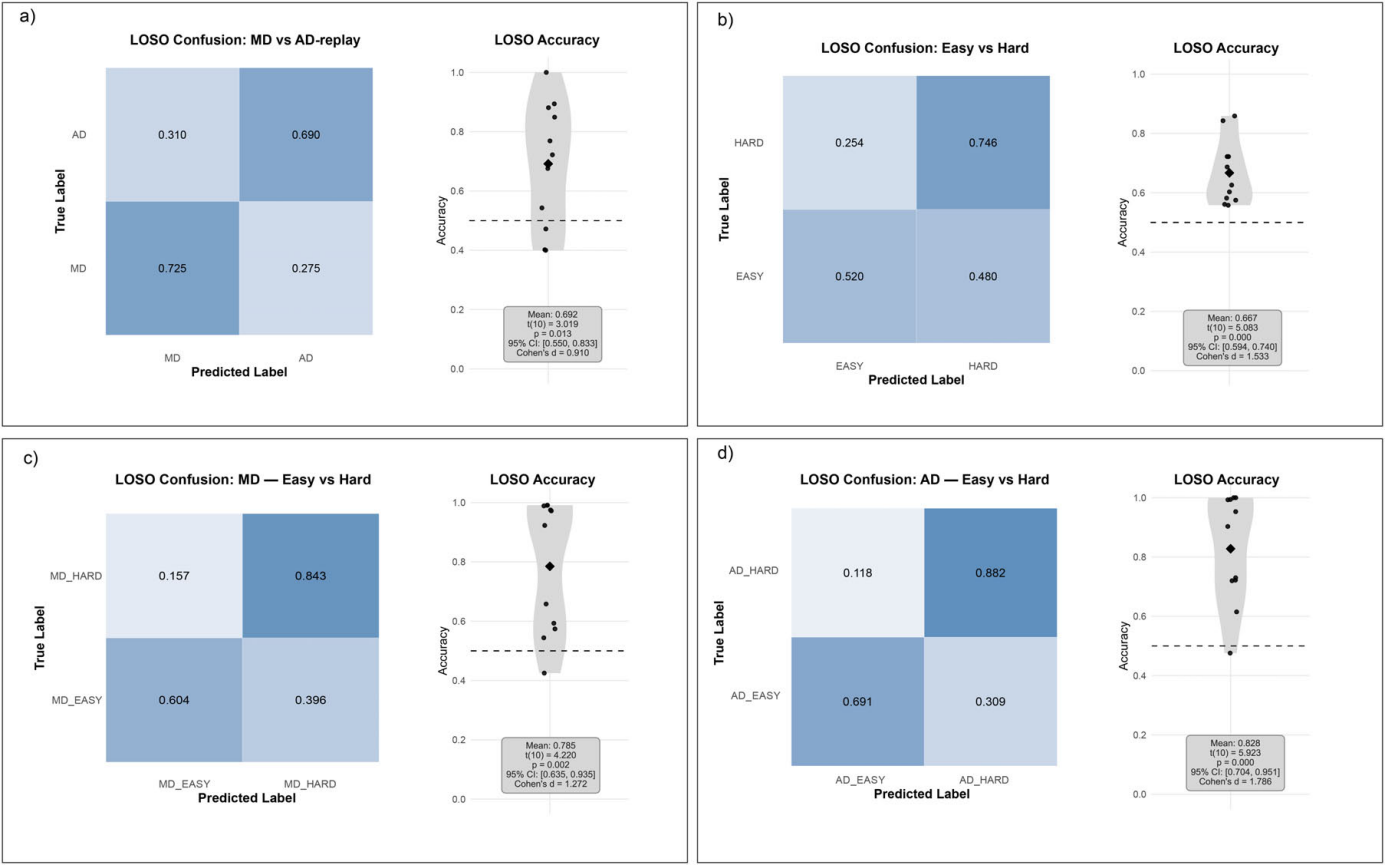
Cross-subject generalization performance using Leave-One-Subject-Out (LOSO) cross-validation. Results are shown for four distinct binary classification tasks: **(a)** engagement mode (Manual Driving vs. AD-replay), **(b)** overall task complexity (Easy vs. Hard), **(c)** complexity within Manual Driving only (MD-Easy vs. MD-Hard), and **(d)** complexity within AD-replay only (AD-Easy vs. AD-Hard). For each task, the left panel displays the group-averaged confusion matrix, normalized by the true label (values on the diagonal represent recall). The right panel shows the distribution of individual subject accuracies from each LOSO fold, presented as a violin and strip plot. The dashed horizontal line indicates the theoretical chance level (50%). Inset text boxes provide the mean accuracy ± standard deviation, and the results of one-sample *t*-tests against chance level, including the t-statistic (t), *p*-value (p), and Cohen’s d effect size.

To rigorously assess the generalizability of the neural signatures, we employed a stringent Leave-One-Subject-Out (LOSO) cross-validation strategy on four distinct binary classification tasks. In all tasks, mean classification accuracy was significantly above the 50% chance level. The results, detailed in Figure 6a–d (confusion matrices, left panels, and violin plots of LOSO accuracy, right panels), revealed a clear hierarchy in generalization performance.

Generalization was most effective when distinguishing task difficulty within a given engagement mode. The classifier achieved a mean accuracy of 82.8% (SD = 18.4%; 95% CI = [0.704, 0.951]; *t*(10) = 5.92, *p* = 0.0001, *d* = 1.79) for classifying difficulty within the AD-replay mode (Figure 6d) and 78.5% (SD = 22.4%; 95% CI = [0.635, 0.935]; *t*(10) = 4.22, *p* = 0.0018, *d* = 1.27) within the MD mode (Figure 6c). Performance was more moderate, though still robust, for the other two tasks: distinguishing between engagement modes (MD vs. AD-replay) yielded a mean accuracy of 69.2% (SD = 21.0%; 95% CI = [0.550, 0.833]; *t*(10) = 3.02, *p* = 0.0129, *d* = 0.91; Figure 6a), and classifying the overall task difficulty across modes yielded a mean accuracy of 66.7% (SD = 10.9%; 95% CI = [0.594, 0.740]; *t*(10) = 5.08, *p* = 0.0005, *d* = 1.53; Figure 6b). Macro-F1 mirrored these patterns (Mode = 0.642 ± 0.307; pooled Difficulty = 0.731 ± 0.105; within-MD = 0.807 ± 0.270; within-AD = 0.846 ± 0.222).

This pattern of results indicates that while the neural signatures of task complexity are relatively consistent across individuals, the signatures differentiating active engagement from passive observation are subject to greater inter-individual variability.

The results of this study delineate the profound reorganization of neural resources that distinguishes active control from passive observation. This distinction was evident across multiple levels of analysis–including source-level activation, spectral dynamics, and large-scale network connectivity–and was further amplified by increasing task complexity. We propose that this multi-level neural reorganization provides a quantitative signature of the cognitive cost of engagement. The robustness of these signatures was underscored by a machine learning model that decoded the operator’s state from single trials with high within-subject accuracy. While the challenge of generalizing across individuals highlights a key avenue for future work, these findings provide a robust empirical foundation for the subsequent discussion, which will unpack their implications for neurocognitive theory and human-machine interaction.

## 4 Discussion

### 4.1 General summary

This study reveals the distinct neurophysiological signatures of active, goal-directed control versus passive observation under complex, matched sensory conditions. Our multi-method EEG analysis demonstrates that active engagement requires a profound reorganization of neural resources, characterized by the recruitment of fronto-parietal executive and attentional networks. This neural distinction, which was amplified by task complexity, was robust enough for machine-learning classification with high within-subject accuracy. We argue that the magnitude of this neural reorganization reflects the inherent cognitive cost of active engagement. Understanding this cost is critical, as it provides a foundational baseline for interpreting the challenges of transitioning between passive and active modes of interaction in complex systems. The following sections will interpret these findings through the lens of neurocognitive theory, examine the study’s methodological contributions, and explore the broader implications of this “cost of engagement” for human-machine interaction.

### 4.2 Functional neuroanatomy: EEG correlates of active control vs. passive observation

The neuroanatomical dissociation we observed between active control and passive observation is consistent with contemporary neurocognitive theories of control, most notably the predictive coding framework (Friston, 2010). Within this framework, active environmental interaction instantiates a process of active inference, where the brain continuously refines its internal models of the world. Our findings of heightened activation in the medial prefrontal cortex (mPFC), anterior cingulate cortex (ACC), and posterior parietal cortex (PPC) during the active state, alongside amplified frontal midline theta, directly map onto the neural substrates of this cognitively demanding process: top-down prediction, error monitoring, and attentional allocation in the face of uncertainty (Cavanagh and Frank, 2014; Corbetta and Shulman, 2002; Klimesch, 1999; Ridderinkhof et al., 2004).

The passive observation condition, by contrast, induced a neural state consistent with predictive quiescence. The observed shift toward altered activity in occipital and medial motor regions, combined with weakened fronto-parietal coupling, suggests the brain defaulted to a passive surveillance mode. This disengagement from active model updating, characterized by diminished executive control signals, closely parallels the neural dynamics seen in other passive observation paradigms (Corbetta and Shulman, 2002; Raichle et al., 2001). Together, these findings highlight that active control and passive observation recruit distinct neuroanatomical systems aligned with divergent computational strategies: goal-directed active control versus prediction-based passive oversight.

While predictive coding explains the underlying computational strategy, Cognitive Load Theory provides a framework for understanding the downstream effects on engagement (Gevins et al., 1998; Wascher et al., 2018). The high workload inherent in active control necessitates the recruitment of executive control networks to manage the cognitive burden–a finding consistent with prior EEG studies reporting elevated frontal theta power under increasing cognitive load (Cavanagh and Frank, 2014; Gevins et al., 1998). Our passive observation condition represents a state of mitigated cognitive load. However, the observed deactivation of the mPFC and ACC in this state may signify a cognitively “hollowed-out” state of engagement – not implying a cessation of processing, but rather a condition in which sensory input is still encoded while higher-order evaluative and error-monitoring functions that normally sustain active control are substantially attenuated. Understanding the neural markers of such a disengaged state is a critical first step for characterizing the cognitive gap that must be overcome during a transition to active control in any high-stakes human-machine system. This challenge is particularly acute in domains like semi-automated driving, where our findings provide a potential framework for understanding the neural basis of safe and effective takeover performance.

And it is also important to distinguish the broadband source activation reported here from the narrowband oscillatory dynamics discussed below. Our finding of greater overall activation in the visual cortex during passive observation (as measured by dSPM) is not in conflict with our finding of greater alpha power during active control. The former likely reflects robust, bottom-up sensory processing of the rich visual stream, while the latter reflects a specific, top-down inhibitory rhythm used to filter that stream for goal-directed action. Together, they paint a more complete picture of the distinct computational strategies employed in each engagement mode.

Several methodological considerations warrant mention. First, the 29-channel montage necessarily provides lower spatial resolution compared with high-density EEG systems. Second, reliance on template anatomy (fsaverage) without individual MRIs introduces co-registration uncertainty on the order of several millimeters. Third, the EEG inverse problem is inherently ill-posed, and thus precise localization cannot be guaranteed. For these reasons, the reported source activations should be interpreted as putative regional estimates rather than definitive loci. Importantly, however, the sensor-space topographies (Supplementary Figure 3) revealed spatial patterns consistent with the source-level contrasts–frontal/occipital enhancements during manual driving, posterior/parietal enhancements during automated replay, and diffuse posterior recruitment under higher task complexity–providing converging evidence independent of inverse modeling assumptions.

### 4.3 Spectral dynamics: the oscillatory fingerprints of engagement

Our spectral analysis reveals that each engagement state is defined by a unique and interpretable “oscillatory fingerprint,” providing a granular, physiologically grounded explanation for the global differences observed between active control and passive observation.

The signature of top-down executive control was unequivocally marked by frontal midline theta (4–8 Hz). Its pronounced enhancement during the active control state, particularly under high cognitive load, aligns perfectly with its established role in conflict monitoring and adaptive regulation in dynamic environments (Botvinick et al., 2001; Cavanagh and Frank, 2014).

**FIGURE 7:**
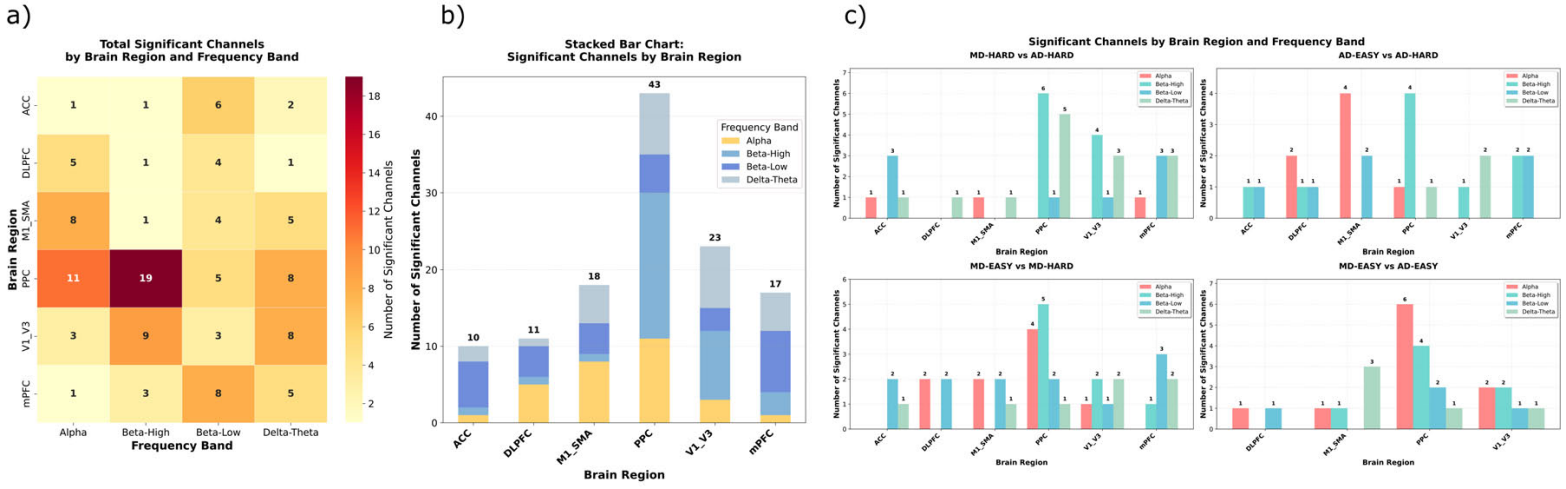
Distribution of frequency-specific EEG differences across brain regions and condition comparisons. **(a)** Heatmap showing the total number of EEG channels exhibiting statistically significant differences across four frequency bands (Alpha, Beta-Low: 13–20 Hz, Beta-High: 20–36 Hz, Delta-Theta: 2–8 Hz) and six functionally defined brain regions (ACC, DLPFC, M1/SMA, PPC, V1–V3, mPFC). The posterior parietal cortex (PPC) exhibited the highest overall count, particularly in the high-beta band. **(b)** Stacked bar plot illustrating the same distribution as panel **(a)**, highlighting the dominant contribution of high-beta and delta-theta differences in PPC and V1–V3, and the multi-band involvement of motor and prefrontal regions. **(c)** Condition-specific bar charts showing the number of significant channels by region and frequency band for each of the four key pairwise comparisons: MD-HARD vs. AD-REPLAY-HARD, AD-REPLAY-EASY vs. AD-REPLAY-HARD, MD-EASY vs. AD-REPLAY-EASY, and MD-EASY vs. MD-HARD. Results demonstrate that high-beta effects dominate PPC during mode-related contrasts (especially MD-HARD vs. AD-REPLAY-HARD), while delta-theta differences are most prominent in mPFC and M1/SMA during complexity-related comparisons (e.g., MD-EASY vs. MD-HARD). These findings support the role of distinct neural oscillatory dynamics in differentiating both task complexity and driving mode.

Concurrently, the state of attentional gating was indexed by occipital alpha power (8–13 Hz). Interestingly, and in contrast to a simple interpretation of visual load, our results indicated greater alpha power (synchronization) during the active control state. This dynamic was most pronounced in the contrast between MD-HARD and AD-REPLAY-HARD within visual and parietal areas (Figure 7c, condition-specific bar plots; see also Figure 7b, stacked bar distribution highlighting alpha contributions in PPC and V1–V3), cleanly separating active visual processing from passive sensory intake. This seemingly paradoxical finding is consistent with the inhibitory gating hypothesis (Jensen and Mazaheri, 2010). Rather than simply reflecting lower visual processing, elevated alpha power can represent an active, top-down neural mechanism for filtering out irrelevant sensory information to prioritize task-relevant cues. In the context of driving, this may reflect a form of “tunnel vision,” where the brain actively suppresses processing of non-essential visual details to focus resources on the road ahead. The concurrent increase in PPC alpha is consistent with strengthened dorsal attention/sensorimotor integration required for steering. Together with elevated frontal-midline theta and motor β/μ modulation, the overall spectral profile aligns with heightened engagement, robustly arguing against a drowsiness or under-arousal account. Although we did not record ocular metrics, matched visual input, artifact-reduced EEG after ICA, and the concurrent, anatomically distributed theta/beta modulations and connectivity changes argue against ocular or illumination factors as the primary driver of the occipito-parietal alpha effect. Still, residual influences cannot be fully excluded. Prior studies show that ocular artifacts typically manifest as frontal topographies with broadband low-frequency activity rather than posterior alpha-band synchronization (Croft and Barry, 2000; Jung et al., 2000). In contrast, our findings of occipito-parietal alpha increases accompanied by frontal-midline theta and motor β/μ dynamics are more consistent with network-level mechanisms of attentional control and inhibitory gating (Cavanagh and Frank, 2014; Engel and Fries, 2010; Jensen and Mazaheri, 2010; Klimesch, 2012). Future work will therefore incorporate eye-tracking and pupillometry to provide direct verification.

The most intriguing findings emerged from the beta band (13–30 Hz), which appeared to track sensorimotor and predictive processes. Its prominent involvement in PPC (19 significant channels in the high-beta range, Figure 7a, heatmap) points to its integral role in spatial processing. Intriguingly, beta’s function appeared to diverge by mode: its desynchronization in motor areas during active control signaled active motor planning (Pfurtscheller and Lopes da Silva, 1999; Pfurtscheller and Neuper, 1997), while its paradoxical increase during high-complexity passive observation may reflect a compensatory state of vigilance or predictive modeling in the absence of physical control. Finally, low-frequency delta (<4 Hz) power increases under the most demanding active-control epochs, consistent with sustained effort in our paradigm.

Collectively, this decomposition reveals a symphony of neural oscillations where each band provides a distinct voice. This rich, multi-band dissociation serves as a detailed neurophysiological accounting of the cognitive cost of engagement, powerfully validating the interpretability of our findings.

### 4.4 Functional connectivity: network-level signatures of engagement

Our analysis of functional connectivity moves beyond regional activity to reveal how large-scale brain networks dynamically reconfigure in response to engagement mode, providing a system-level perspective on the neurocognitive dichotomy between active control and passive observation. The defining feature of active control was a robust increase in long-range fronto-parietal coherence, predominantly in the theta and low-beta bands. This synchronized dialogue between medial frontal and posterior parietal sites is the hallmark of integrated top-down control, aligning with established accounts of fronto-parietal control networks (Corbetta and Shulman, 2002; Miller and Cohen, 2001). The amplification of theta coherence under high cognitive load underscores its role in error monitoring and adaptive control (Cavanagh and Frank, 2014; Cohen, 2011), while low-beta synchrony likely orchestrates the continuous interplay between sensorimotor planning and spatial attention (Engel and Fries, 2010; Kilavik et al., 2013; Spitzer and Haegens, 2017).

Conversely, passive observation was characterized by a state of network segregation. The attenuation of fronto-parietal coherence signified a decoupling of executive and attentional systems. In its place, connectivity became more localized within a posterior visual-parietal network–a neural architecture seemingly optimized for passive visual surveillance rather than active intervention. This shift toward a more modular, less integrated state finds strong parallels with findings on passive vigilance and the dynamics of the Default Mode Network during periods of low cognitive demand (Raichle et al., 2001; Sadaghiani and D’Esposito, 2015).

Significantly, this network-level narrative was not merely a statistical observation but also a key element in the decodability of the operator’s state. The remarkable convergence between our coherence maps and the feature importance topographies from the machine learning model provides powerful, data-driven validation. The prioritization of these exact fronto-parietal connectivity metrics by the classifier corroborates that the temporal synchronization between distributed cortical hubs is a definitive signature of an actively engaged state.

In essence, our findings indicate that the transition from active control to passive observation is not merely a change in local brain activity, but a fundamental reorganization of the brain’s functional architecture. This architectural shift from a segregated, monitoring-oriented state to a highly integrated network for adaptive regulation represents the systems-level manifestation of the cognitive cost of engagement. This insight emphasizes that network-based metrics are indispensable for creating truly comprehensive and reliable models of cognitive state.

### 4.5 Inter-individual variability, generalizability, and limitations

While our study revealed robust group-level neural effects, translating these findings into reliable monitoring systems hinges on addressing the challenge of inter-individual variability. Our Leave-One-Subject-Out (LOSO) cross-validation results quantitatively illustrate this point. Generalization was strongest for task complexity within a given engagement mode–AD: 0.828 and MD: 0.785 accuracy on average (Figure 6c, d). When difficulty was pooled across modes, performance was moderate (mean ≈ 0.667) (Figure 6b). Discriminating engagement mode (MD vs. AD) generalized less well than the within-mode difficulty contrasts, but remained clearly above chance (mean ≈ 0.692) (Figure 6a). This pattern suggests that while complexity-related neural signatures are comparatively stable across individuals, the expression of volitional engagement is more subject-specific. Such variability–well documented in the EEG literature–likely reflects differences in cognitive capacity, task expertise, vigilance, and neuroanatomy, and may be further compounded by the sparse spatial resolution of our EEG system.

**FIGURE 8:**
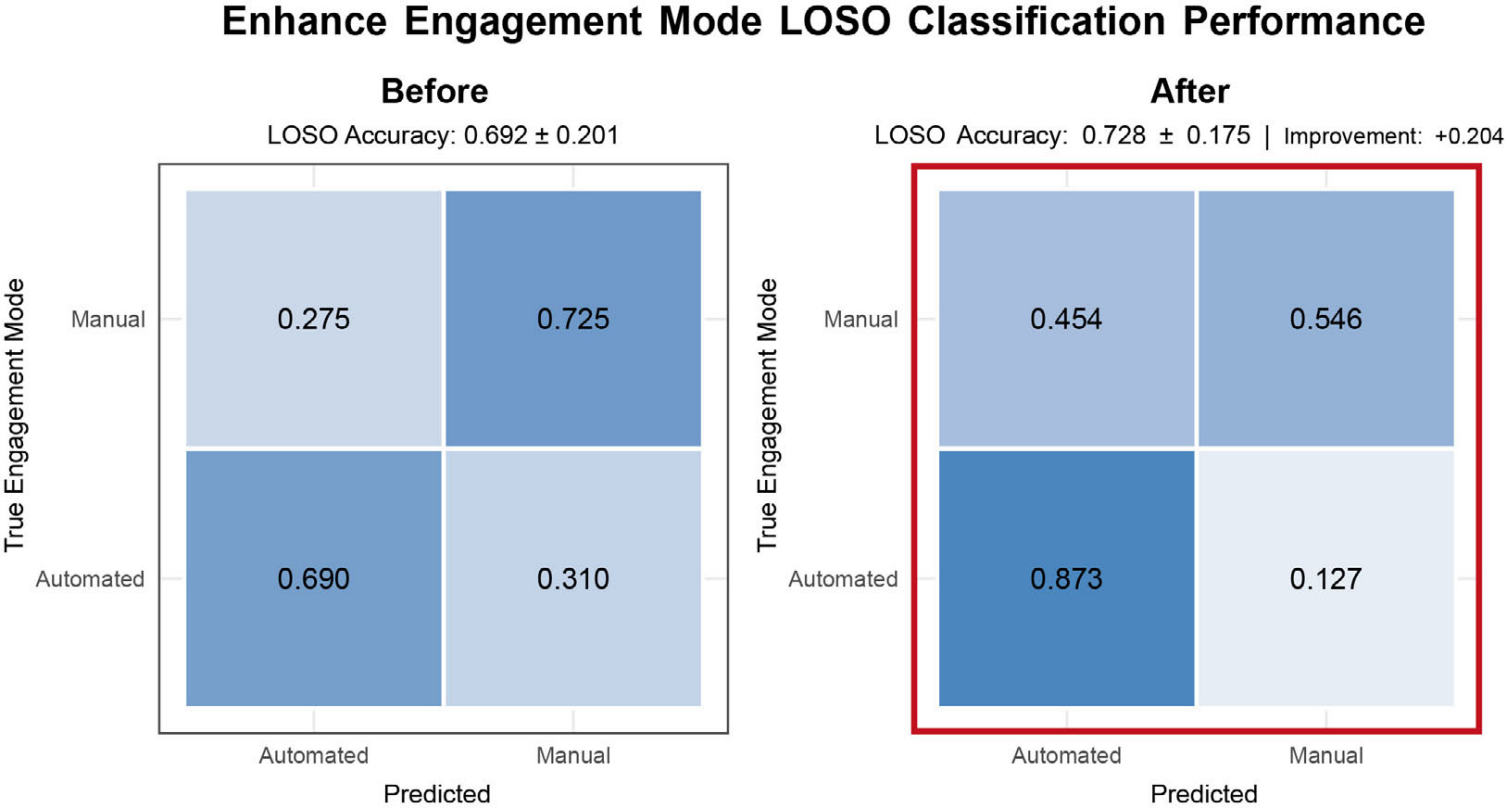
Enhancement of LOSO classification performance for engagement mode. Confusion matrices show improved accuracy from baseline (left: accuracy = 69.2%) to optimized feature configuration (right: accuracy = 72.8%), demonstrating the practical potential for methodologically-driven generalizability enhancement.

It is important, however, to contextualize these generalization challenges within the primary aims of our study. In this research, our focus was not to develop a universally generalizable classifier, but rather to uncover consistent, interpretable neural signatures of distinct engagement states. Nevertheless, to demonstrate the potential for overcoming this variability, we conducted a preliminary model optimization focused on the most challenging task: mode classification. This procedure followed a systematic three-stage approach: (1) domain-driven feature engineering emphasized task-relevant EEG markers such as μ/β rhythms over motor cortex, frontal theta–alpha ratios, and motor asymmetry indices; (2) feature selection and preprocessing employed robust scaling and mutual-information–based selection to reduce dimensionality while mitigating subject variability; and (3) sample-adaptive model selection adjusted classifier complexity across LOSO folds depending on training set size, balancing robustness and flexibility. As illustrated in Figure 8 (left: baseline model, right: optimized model), this resulted in a modest increase in overall mean LOSO accuracy from 69.2% to 72.8%. However, a closer inspection of the confusion matrices reveals a crucial performance trade-off. The optimized model’s ability to correctly identify the AD-replay state improved dramatically [Figure 8, right; recall increased from 69.0% in the baseline (Figure 8, left) to 87.3%], largely by reducing instances where it was mistaken for the MD state. This gain came at the cost of reduced accuracy for identifying the MD state (recall decreased from 72.5% in Figure 8, left to 54.6% in Figure 8, right). This suggests our theory-driven features are highly effective at capturing the consistent neural markers of the passive state across individuals. Ultimately, this result serves as a crucial proof-of-concept, demonstrating that targeted feature engineering can reshape and improve model generalizability, even while highlighting the distinct challenges of classifying different cognitive states.

Crucially, beyond the issue of generalizability, our findings must be interpreted within the context of several other methodological limitations. First and foremost, we must emphasize that the AD-replay condition was designed as a low-engagement baseline and is not a direct simulation of the supervisory control found in real-world automated systems (e.g., SAE Level 2/3). This distinction necessarily limits the immediate applicability of our findings to interactive driving scenarios. However, this trade-off between ecological validity and experimental control was a deliberate methodological choice. By creating a “zero-engagement” state with matched visual input, our design allowed us to isolate the fundamental neural signatures of active engagement with high precision. This rigorously established baseline is therefore not an endpoint, but a crucial first step, providing a solid foundation upon which future studies can build to investigate more complex and realistic supervisory states.

The remaining limitations, such as the fixed experimental order, the modest sample size, and the use of a simulator, also call for caution when generalizing our findings, given the known influence of real-world factors such as perceived risk and situational urgency (Greenlee et al., 2018; McDonnell et al., 2023; Parasuraman and Riley, 1997). Specifically, the manual driving session always preceded the replay condition, since individualized replay stimuli could only be generated after active driving and track familiarity was necessary for participants to adopt a supervisory stance. To reduce potential order-related confounds, we incorporated a 10-min rest between sessions and focused our analyses on within-subject contrasts with identical visual input.

Nevertheless, residual influences of time-on-task, vigilance decline, or expectancy cannot be entirely excluded, and our conclusions are therefore framed conservatively as differences between active manual control and a passive replay baseline. Although participants were instructed to adopt a supervisory stance during replay to maintain attentional engagement, this framing served only as an experimental instruction. The AD-replay condition does not replicate real-world automated driving, which entails supervisory authority and the possibility of takeover. Accordingly, we interpret AD-replay strictly as a passive replay baseline, while broader implications for supervisory automation are reserved for future research. In future work, we also plan to incorporate subjective fatigue questionnaires (and potentially complementary physiological indices) to more directly assess vigilance changes across conditions, thereby further mitigating this potential confound. Nevertheless, we acknowledge that without direct ocular or pupillometric recordings, residual influences of eye dynamics or vigilance cannot be fully excluded. Future work will therefore incorporate eye-tracking and pupillometry alongside subjective fatigue measures to provide stronger control over these factors.

In addition, given the potential concern that differences in motor output and posture could introduce residual EMG and ocular artifacts, we conducted quantitative ICA/ICLabel evaluations and robustness checks. As summarized in Supplementary Table 1, the number of eye- and muscle-related independent components was comparable between MD and AD-replay sessions, with MD in fact showing numerically fewer artifacts. Supplementary Table 2 further demonstrates that excluding peripheral and motor-related electrodes or adopting stricter ICLabel thresholds (0.5–0.9), did not alter the classification results in any substantive way. These analyses indicate that the observed neural differences are unlikely to be explained by artifact contamination alone. Nevertheless, without concurrent EMG or eye-tracking data, residual influences cannot be entirely ruled out, and our conclusions should therefore be interpreted with this caveat in mind.

### 4.6 Implications and future directions

The primary contribution of this research is the neurophysiological characterization of the cognitive cost of active engagement. By demonstrating the profound differences in neural dynamics between active control and passive observation under matched sensory input, we provide a foundational baseline for understanding why transitioning between these states is non-trivial. This “cost of engagement” has critical implications for any domain where humans are required to shift from a role of passive monitoring to one of active intervention, including aviation, industrial process control, and medical supervision.

Building on this baseline, future research can formulate more precise hypotheses. For instance, we hypothesize that an interactive, supervisory task–requiring intermittent monitoring and potential intervention–would elicit neural signatures falling between the two poles we have established. Quantifying how different system designs or interfaces modulate this “engagement gap” is a critical next step.

However, translating these findings into reliable real-world applications requires overcoming the challenge of inter-subject variability, particularly in the neural signatures of engagement mode, which our LOSO results identified as being highly subject-specific in contrast to the more generalizable signatures of task complexity. Future work should therefore explore subject-invariant modeling approaches, such as advanced deep learning architectures and domain adaptation techniques, which represent a critical step toward creating robust, neuroadaptive interfaces.

## 5 Conclusion

In conclusion, by methodologically isolating the mode of engagement, employing a matched-stimulus replay paradigm in a complex driving task, this study delineated the profound reorganization of neural resources that distinguishes active control from passive observation. These findings establish a rigorous neurophysiological baseline for the cognitive cost of engagement, providing essential groundwork for understanding and designing effective human-machine systems where the seamless transition between observation and action is critical for safety and performance.

## Data Availability

The raw data supporting the conclusions of this article will be made available by the authors, without undue reservation.
