# Supplementary material for: Neural signatures of engagement in driving: comparing active control and passive observation
Source: Front Neurosci. 2025 Nov 6;19:1698625. doi: 10.3389/fnins.2025.1698625 (PMC12631349; doi:10.3389/fnins.2025.1698625)
Supplement: Supplementary file 1 [file Data_Sheet_1.docx]

**Supplementary materials**


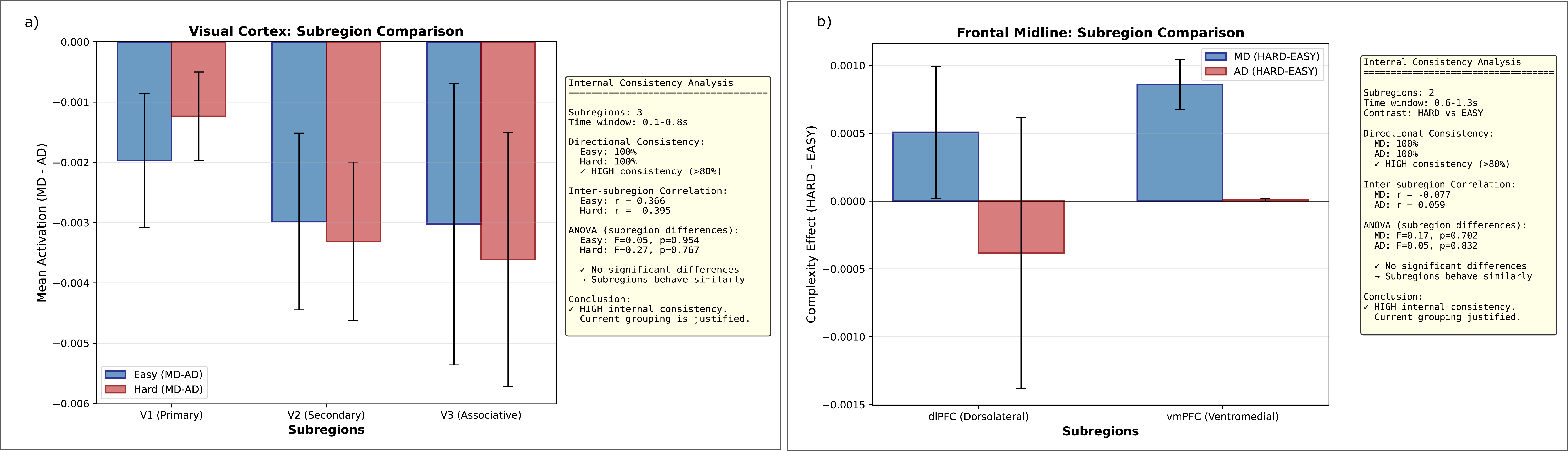


**Fig. S 1 Subregional consistency analyses for ROI validation.** (a) Visual cortex (V1–V3): MD vs. AD-replay contrasts revealed consistent activation trends across subregions. (b) Frontal midline (dlPFC vs. vmPFC): Subregions showed comparable responses to task complexity under both engagement modes.





**Fig. S 2 Per-fold calibration and confusion analyses under the leakage-safe evaluation.** Top row: **reliability (calibration) curves** for the four classes (MD-EASY, MD-HARD, AD-EASY, AD-HARD) computed on the **held-out split of each fold** in a **5-fold GroupKFold** with **segment/lap-level grouping**. Markers correspond to **10 uniform probability bins**; the dashed line denotes **perfect calibration**. Bottom row: the corresponding **row-normalized confusion matrices** for each held-out fold (values are per-class recalls); the panel titles report the **fold accuracies** (~0.87–0.92).





**Fig. S 3 Sensor-space topographies for all contrasts.** Grand-average scalp voltage maps illustrating condition contrasts across successive post-stimulus latencies (150–1100 ms; columns). Rows correspond to different contrasts: (top) manual driving (MD) minus automated replay (AD) under Easy segments, (second row) MD minus AD under Hard segments, (third row) Hard minus Easy within MD, and (bottom row) Hard minus Easy within AD. Warm colors (red) indicate relatively higher amplitudes for the first condition in each contrast, whereas cool colors (blue) indicate higher amplitudes for the second condition. These sensor-level topographies qualitatively corroborate the source-space results reported in the main text, showing frontal/occipital enhancements during MD compared with AD, and more posterior–parietal enhancements during AD as well as diffuse posterior recruitment under Hard conditions in AD relative to MD. Color scale reflects voltage amplitude in μV.

| Condition and participant id | Retained (Brain ≥ 0.5) component number and rate | | Retained (Brain ≥ 0.6) component number and rate | | Retained (Brain ≥ 0.7) component number and rate | | Retained (Brain ≥ 0.8) component number and rate | | Retained (Brain ≥ 0.9) component number and rate | | Brain | | Eye | Muscle | | Other |
| --- | --- | --- | --- | --- | --- | --- | --- | --- | --- | --- | --- | --- | --- | --- | --- | --- |
| MD-S0 | 15 | 51.70% | 10 | 34.50% | 8 | 27.60% | **7** | **24.10%** | 4 | 13.80% | 15 | 1 | | 0 | 13 | |
| AD-S0 | 10 | 34.50% | 6 | 20.70% | 4 | 13.80% | **4** | **13.80%** | 3 | 10.30% | 13 | 3 | | 0 | 13 | |
| MD-S1 | 12 | 41.40% | 11 | 37.90% | 9 | 31.00% | **4** | **13.80%** | 4 | 13.80% | 15 | 3 | | 1 | 10 | |
| AD-S1 | 10 | 34.50% | 8 | 27.60% | 7 | 24.10% | **6** | **20.70%** | 5 | 17.20% | 14 | 3 | | 3 | 9 | |
| MD-S2 | 10 | 34.50% | 10 | 34.50% | 4 | 13.80% | **4** | **13.80%** | 3 | 10.30% | 11 | 4 | | 0 | 14 | |
| AD-S2 | 9 | 31.00% | 9 | 31.00% | 7 | 24.10% | **6** | **20.70%** | 5 | 17.20% | 11 | 3 | | 2 | 13 | |
| MD-S3 | 10 | 34.50% | 9 | 31.00% | 8 | 27.60% | **6** | **20.70%** | 3 | 10.30% | 12 | 1 | | 0 | 16 | |
| AD-S3 | 10 | 34.50% | 7 | 24.10% | 5 | 17.20% | **5** | **17.20%** | 4 | 13.80% | 13 | 3 | | 0 | 13 | |
| MDS4 | 14 | 48.30% | 13 | 44.80% | 9 | 31.00% | **8** | **27.60%** | 6 | 20.70% | 19 | 1 | | 0 | 9 | |
| AD-S4 | 10 | 34.50% | 10 | 34.50% | 9 | 31.00% | **7** | **24.10%** | 4 | 13.80% | 12 | 3 | | 2 | 12 | |
| MD-S5 | 10 | 34.50% | 9 | 31.00% | 7 | 24.10% | **6** | **20.70%** | 2 | 6.90% | 12 | 4 | | 2 | 11 | |
| AD-S5 | 10 | 34.50% | 8 | 27.60% | 7 | 24.10% | **4** | **13.80%** | 4 | 13.80% | 12 | 4 | | 1 | 12 | |
| MD-S6 | 14 | 48.30% | 13 | 44.80% | 10 | 34.50% | **9** | **31.00%** | 5 | 17.20% | 20 | 2 | | 0 | 7 | |
| AD-S6 | 8 | 27.60% | 6 | 20.70% | 6 | 20.70% | **4** | **13.80%** | 4 | 13.80% | 14 | 2 | | 0 | 13 | |
| MD-S7 | 11 | 37.90% | 10 | 34.50% | 10 | 34.50% | **7** | **24.10%** | 4 | 13.80% | 13 | 2 | | 4 | 10 | |
| AD-S7 | 6 | 20.70% | 5 | 17.20% | 4 | 13.80% | **4** | **13.80%** | 3 | 10.30% | 8 | 3 | | 6 | 12 | |
| MD-S8 | 7 | 24.10% | 4 | 13.80% | 4 | 13.80% | **3** | **10.30%** | 3 | 10.30% | 9 | 3 | | 0 | 17 | |
| AD-S8 | 11 | 37.90% | 11 | 37.90% | 11 | 37.90% | **10** | **34.50%** | 5 | 17.20% | 14 | 2 | | 0 | 13 | |
| MD-S9 | 8 | 27.60% | 7 | 24.10% | 2 | 6.90% | **2** | **6.90%** | 2 | 6.90% | 12 | 1 | | 3 | 13 | |
| AD-S9 | 8 | 27.60% | 5 | 17.20% | 5 | 17.20% | **5** | **17.20%** | 3 | 10.30% | 9 | 1 | | 3 | 16 | |
| MD-S10 | 17 | 58.60% | 14 | 48.30% | 11 | 37.90% | **8** | **27.60%** | 5 | 17.20% | 17 | 3 | | 1 | 8 | |
| AD-S10 | 10 | 34.50% | 10 | 34.50% | 10 | 34.50% | **9** | **31.00%** | 6 | 20.70% | 11 | 3 | | 1 | 14 | |
| Average | 10.5 | 36.34% | 8.8 | 30.56% | 7.1 | 24.61% | **5.8** | **20.06%** | 3.95 | 13.64% | 13.1 | 2.5 | | 1.3 | 12.1 | |

**Table S 1 ICA Component Classification Summary.** Summary of independent component (IC) classifications obtained with ICLabel across Manual Driving (MD) and Automated Driving–Replay (AD) conditions. Values represent the mean ± standard deviation of components labeled as ocular (eye movements), muscular (EMG), and other artifact categories per subject, together with the proportion of retained components at multiple decision thresholds (0.5–0.9). These results demonstrate that MD did not yield systematically more artifact components than AD, and that ICA effectively normalized artifact levels between conditions.

| **Condition** | **Mean Accuracy** | **Std Dev (Acc)** | **Mean F1-Score** | **Std Dev (F1)** | **Retention Rate (%)** |
| --- | --- | --- | --- | --- | --- |
| **ICA 80% Threshold** | **0.8986** | **0.0337** | **0.8182** | **0.0358** | **20.06%** |
| Without_ica (raw) | 0.6729 | 0.0402 | 0.663 | 0.0394 | N/A |
| ICA 50% Threshold | 0.8117 | 0.0228 | 0.8062 | 0.0247 | 36.05% |
| ICA 60% Threshold | 0.7869 | 0.0337 | 0.7779 | 0.0339 | 30.56% |
| ICA 70% Threshold | 0.8144 | 0.0242 | 0.8112 | 0.0234 | 24.61% |
| ICA 90% Threshold | 0.8451 | 0.0174 | 0.8423 | 0.017 | 13.64% |
| **Exclude Peripheral Electrodes** | **0.8893** | **0.0189** | **0.8865** | **0.0182** | **N/A** |
| **Exclude Motor Electrodes** | **0.8966** | **0.013** | **0.8944** | **0.0143** | **N/A** |

**Table S 2 Classifier Robustness under Different ICA Retention Thresholds.** Classification accuracy (mean ± standard deviation) of the Manual Driving (MD) vs. Automated Driving–Replay (AD) four-class model under progressively stricter ICA retention thresholds (0.7–0.9). The proportion of retained components is indicated for each threshold. Results show that even with aggressive artifact removal (threshold = 0.9, retaining only 13.6% of components), classification performance remained substantially higher than the no-ICA baseline, confirming that findings are robust to stringent artifact rejection.

| ROI / Frequency | Hypothesis | Condition | Prior Prediction | Reference | Original Mean | Original p | Bootstrap Mean (95% CI) | Bootstrap p (FDR) | Sig. | Notes |
| --- | --- | --- | --- | --- | --- | --- | --- | --- | --- | --- |
| mPFC (activation) | H1: Mode (MD > AD) | Hard | ↑ Executive control | Miller & Cohen (2001) | 0.0065 | <0.001 | -0.0016 (−0.0048, ∞) | 0.768 (FDR) | **No** | Orig. sig., bootstrap variable |
| ACC (activation) | H1: Mode (MD > AD) | Hard | ↑ Conflict monitoring | Botvinick et al. (2001) | 0.012 | <0.001 | 0.0019 (−0.0035, ∞) | 0.354 (FDR) | **No** | Orig. sig., bootstrap inconsistency |
| Motor (activation) | H1: Mode (AD > MD trend) | Easy / Hard | None | N/A | -0.0017 / -0.0018 | 0.627 / 0.560 | -0.0009 (−0.0016, −0.0001) | 0.016 (uncorr.) | **Trend** | Exploratory only |
| PPC (activation) | H1: Mode (MD > AD) | Easy / Hard | ↑ Spatial attention | Wascher et al. (2018) | 0.0036 / 0.0035 | 0.001 / <0.001 | 0.0023 (−0.0019, 0.0063) | 0.293 (uncorr.) | **No** | Effect did not replicate |
| DLPFC (activation) | H1: Mode | Merged | – | – | 0.003 | 0.055 | 0.003 (−0.00003, 0.0064) | 0.055 (uncorr.) | **Marginal** | Trend only |
| mPFC Theta | H1: Mode (MD > AD) | Easy / Hard | ↑ Cognitive control | Cavanagh & Frank (2014) | – | 0.011 / 0.004 | 1.6–1.9e−5 (6.9e−6, ∞) | <0.001 (FDR) | **Yes** | Robust |
| ACC Theta | H1: Mode (MD > AD) | Easy / Hard | ↑ Conflict monitoring | Cavanagh & Frank (2014) | – | 0.004 / <0.001 | 2.4–2.6e−5 (6.3e−6, ∞) | <0.001 (FDR) | **Yes** | Robust |
| Motor Mu | H1: Mode (MD > AD, ERD) | Easy / Hard | ↑ Sensorimotor integration | Pfurtscheller & Lopes da Silva (1999) | -50.1% ERD | 0.019 | 1.2–2.4e−5 (7.1e−6, ∞) | <0.001 (FDR) | **Yes** | Robust |
| Motor Beta | H1: Mode (MD > AD) | Hard | ↑ Motor prep. | Pfurtscheller & Neuper (1997) | – | 0.047 | 5.0e−5 (2.9e−5, ∞) | <0.001 (FDR) | **Yes** | Robust |
| Visual Alpha | H1: Mode (MD > AD) | Easy / Hard | ↑ Visual attention | – | – | 0.001 / <0.001 | 1.7–4.8e−5 (8.4e−6, 1.2e−4) | <0.001 (uncorr.) | **Trend** | Consistent exploratory effect |
| PPC Alpha | H1: Mode (MD > AD) | Easy / Hard | ↑ Spatial attention | – | – | 0.018 / 0.004 | 6.7–16.8e−6 (2.6e−6, 4.5e−5) | <0.001 (uncorr.) | **Trend** | Consistent exploratory effect |
| mPFC Theta | H2: Complexity (Hard > Easy, MD) | MD | ↑ Cognitive demand | Tang et al. (2022) | – | – (qual.) | 2.1e−5 (6.8e−6, ∞) | <0.001 (uncorr.) | **Yes** | Confirmed bootstrap |
| ACC Theta | H2: Complexity (Hard > Easy, MD) | MD | ↑ Conflict monitoring | Cavanagh & Frank (2014) | – | – (qual.) | 3.9e−5 (1.6e−5, ∞) | <0.001 (uncorr.) | **Yes** | Confirmed bootstrap |
| ACC Theta | H2: Complexity (Hard vs Easy, AD) | AD | – | – | – | – (qual.) | 4.1e−5 (1.2e−5, 1.1e−4) | <0.001 (uncorr.) | **Trend** | Consistent exploratory effect |

**Table S 3 Detailed statistical outcomes of ROI- and frequency-level analyses.** The table reports (i) original test results (test type, mean difference, p-value, and nominal significance), (ii) bootstrap resampling estimates (5,000 iterations; mean effect size, 95% confidence interval, and bootstrap p-values), and (iii) false discovery rate (FDR)–adjusted significance within each hypothesis family. For exploratory contrasts, results are shown descriptively as trends. This table provides the full inferential context underlying the summary statistics presented in the main text (Sections 3.1–3.2), where H1: Hypothesis 1; H2: Hypothesis 2; MD = manual driving; AD = automated driving (passive replay); ROI = region of interest; ERD/ERS = event-related desynchronization/synchronization; Bootstrap Mean (95% CI) = resampled group mean with 95% confidence interval from 5,000 bootstrap iterations; Bootstrap p = bootstrap-derived p-value; Bootstrap p (FDR) = p-value after false discovery rate correction (Benjamini–Hochberg) within each family of tests; Bootstrap Significant = indicates whether the effect remained significant after bootstrap and correction; Trend (uncorrected) = effects that did not survive correction but showed consistent directionality; – (qual.) = qualitative description in the original manuscript without formal statistical test
