# Supplementary figures and images for: Neural signatures of engagement in driving: comparing active control and passive observation
Source: Front Neurosci. 2025 Nov 6;19:1698625. doi: 10.3389/fnins.2025.1698625 (PMC12631349; doi:10.3389/fnins.2025.1698625)

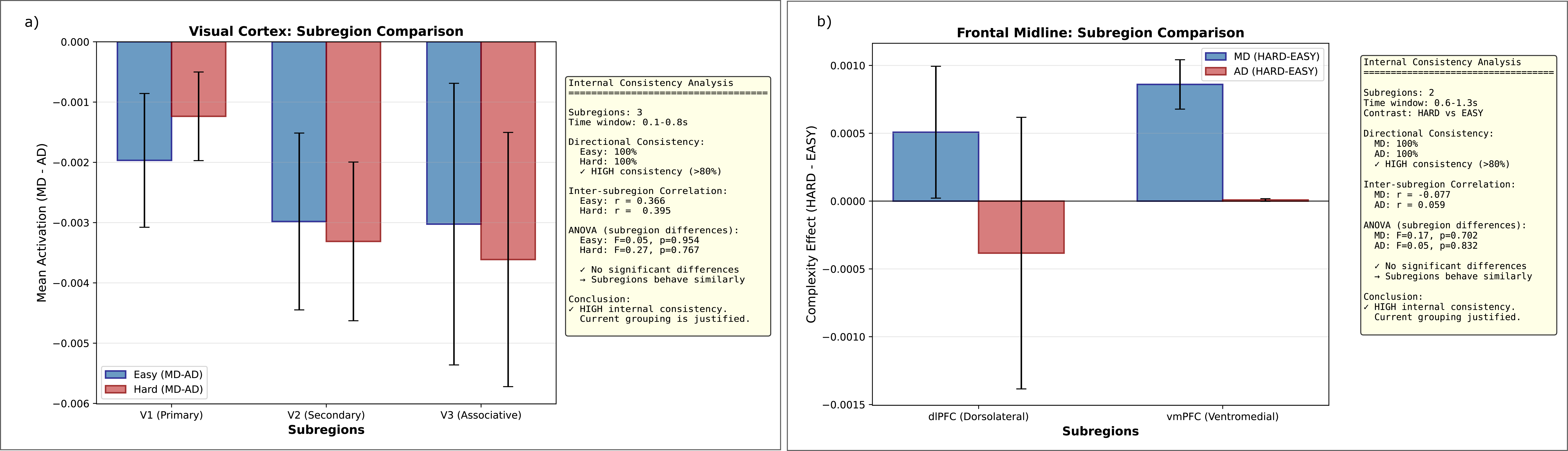

Supplement: Supplementary file 2 [file Image_1.jpeg]
